# Rapamycin Partially Reverts Cavernoma Endothelial Cell Phenotype and, When Combined With Lapatinib, Ameliorates Chronic Lesions

**DOI:** 10.1111/jcmm.71280

**Published:** 2026-07-09

**Authors:** Mar García‐Colomer, José E. Martínez, Luis Díaz‐Gómez, Miriam Sartages, Eva M. Esquinas‐Román, Cristina Riobello, David Martínez‐Delgado, Diego González‐Pérez, Aurora Gómez‐Durán, Miguel Fidalgo, Marta Varela‐Rey, Celia M. Pombo, Juan Zalvide

**Affiliations:** ^1^ Department of Physiology, Centro Singular De Medicina Molecular E Enfermedades Crónicas (CiMUS) and Instituto Sanitario De Santiago De Compostela (IDIS) Universidade De Santiago De Compostela (USC) Santiago de Compostela A Coruña Spain; ^2^ Department of Pharmacology, Pharmacy, and Pharmaceutical Technology Instituto De Materiales (iMATUS), and Instituto Sanitario De Santiago De Compostela (IDIS), Universidade De Santiago De Compostela Santiago de Compostela A Coruña Spain; ^3^ Department of Biochemistry and Molecular Biology, Centro Singular De Medicina Molecular E Enfermedades Crónicas (CiMUS) and Instituto Sanitario De Santiago De Compostela (IDIS) Universidade de Santiago de Compostela (USC) Santiago de Compostela A Coruña Spain

**Keywords:** cerebral cavernous malformations, Lapatinib, PDCD10, Propranolol, Rapamycin

## Abstract

This study investigates the impact of rapamycin and propranolol on cerebral cavernous malformations (CCMs). Employing an unbiased transcriptomic analysis, we aimed to comprehensively elucidate the molecular mechanisms underlying these drug effects. Mouse Brain Microvascular Endothelial Cells (mBMEC) deficient in *Ccm3* were treated with propranolol or rapamycin and were analysed by RNA‐seq and immunofluorescence. While propranolol shows limited efficacy in modulating the CCM transcriptomic phenotype in mBMEC, rapamycin demonstrates a significant impact. Rapamycin partially reverses gene expression changes induced by *Ccm3* deficiency, restoring KLF2/4‐dependent genes like *Nos3*, *Adamts1*, and *Thbs1*. Notably, we observed a reduction in KLF2 protein levels in *Ccm3* KO cells treated with rapamycin. We also sought to determine whether rapamycin, especially in combination with the tyrosine kinase inhibitor lapatinib, which induces proapoptotic gene expression in *Ccm3*‐deficient endothelium, can reduce lesion volume even after lesion growth has occurred. *Ccm3* ^iEC^ mice in which cavernoma development had been induced were treated with rapamycin alone or combined with lapatinib, assessing lesion volume using micro‐CT imaging. Notably, a combination of rapamycin and lapatinib effectively reduces lesion volume in a chronic CCM model. In summary, our work reveals a mechanism by which rapamycin modulates *Ccm3* KO endothelial cells and identifies rapamycin plus lapatinib as a possible combination therapy for cavernomas.

## 1 Introduction

Cerebral cavernous malformations (CCMs) are common cerebral vascular malformations [1, 2] predominantly occurring in the central nervous system and associated with recurrent haemorrhages and seizures [3, 4]. Most CCMs are sporadic, involving somatic mutations, but they can also have an inherited genetic basis caused by inactivating mutations in one of the three CCM genes: *KRIT1/CCM1*, *OSM/CCM2* and *PDCD10/CCM3* [2]. Patients with this familial cavernomatosis (fCCM) have multiple CCMs, which are associated with greater lesion burden and clinical severity. The products of the three CCM genes form a ternary complex that regulates endothelial differentiation, permeability, *Vegfr2* and *Egfr* expression, exocytosis, HEG1 receptor activity, β‐catenin and VE‐cadherin distribution, RHO/ROCK signalling and stress fibre formation, ERK5 phosphorylation and downstream KLF2/KLF4 activity, and inhibition of endothelial‐to‐mesenchymal transition [4]. Among these processes, dysregulation of KLF2 and KLF4 in CCM mutant cells is essential for cavernoma development [5].

The current treatment for CCMs relies on surgical intervention for accessible lesions in the brain and spinal cord. However, for deep‐seated CCM lesions and for fCCMs, when many lesions may appear, novel pharmacological interventions are clearly needed, and their development is a long‐standing goal of translational CCM research. Several possible drug treatments have been proposed, among them statins, known to inhibit Rho activity, which is deregulated in CCM‐deficient cells, especially in *Ccm1*‐ and *Ccm2*‐deficient endothelium [6, 7]; sulindac, which inhibits transcriptional β‐catenin activity [8]; the multikinase inhibitor sorafenib, which inhibits excessive angiogenesis [9]; the free radical scavenger tempol [10]; intestinal microbiome modification to alter Toll‐mediated signalling [11]; vitamin D and indirubin‐3‐monoxime, identified in a systematic pharmacological screen [12]; lapatinib, because of the increase of EGFR in *Ccm3*‐deficient endothelial cells [13]; or rapamycin, based on the stimulated mTORC1 activity in CCM lesions and autophagy defects in CCM‐deficient cells [14, 15, 16, 17]. All these treatments diminish one or several of the effects of CCM deficiency in vitro, and some of them diminish the number of lesions that arise in animal models of cavernoma development when given before their appearance or growth. Despite promising preclinical results, no clinically significant effect has been found in the two trials performed so far, a pilot study evaluating the effects of simvastatin on lesion permeability [18] and a blind study on the effects of atorvastatin on lesion bleeding [19]. Also, the phase 2 trial using the drug REC‐994 (NCT05085561) has recently been discontinued because of a lack of clinical benefits.

Interestingly, the largest number of clinical trials proposed for CCMs involve the non‐selective β‐blocker propranolol (trials NCT03589014, NCT03474614 and NCT03523650), which has been selected due to its known effect on infantile haemangiomas [20]. Another treatment that has been proposed recently is rapamycin. This was initially based on the enhanced mTORC1 activity in CCM‐deficient cells [17] and the seminal observation that *Pik3ca* gain‐of‐function (GOF) mutations fueled the growth of cavernoma lesions, reinforced by the fact that CCM‐deficient endothelial cells without *PIK3CA* mutations also had increased phosphorylation of the mTORC1 target S6 [14].

Propranolol treatment has been shown to reduce lesion burden in chronic murine models of cavernoma development and restore barrier function when initiated before or during lesion growth [21]. Furthermore, while meta‐analyses of clinical studies have not demonstrated a statistically significant protective effect of β‐blockers (including propranolol) in preventing intracerebral haemorrhage or focal neurologic deficits in individuals with CCM, a phase II clinical trial suggested a potential benefit in preventing cavernoma development, although the study was not powered to achieve statistical significance [22]. Despite these indications of activity, the clinical efficacy of propranolol remains unclear. Clarifying this uncertainty requires further work to define the cellular and molecular mechanisms of propranolol in CCM, including the specific cell type in which it exerts its effects. Several studies have described the effects of propranolol on CCM‐deficient endothelial cells, but at a concentration of 100 μM [23], which is significantly higher than the estimated in vivo concentration after propranolol treatment, even in the brain (not exceeding approximately 10 μM). Moreover, treatment of mice with 15 mg/kg.day of propranolol did not affect lesion size or haemorrhage in already established cavernomas, at least under the treatment regimen used [24].

As stated above, the combination of the detection of *Pik3ca* GOF mutations in large cavernomas and enhanced mTORC1 activity in CCM‐deficient cells makes mTORC1‐inhibiting drugs such as rapamycin good candidates for pharmacological treatment of cavernomas. Enhanced activity of mTORC1 has been shown in *CCM*‐deficient endothelial cells [14, 17] and in progenitor endothelial cells in in vivo lesions [25]. Its inhibition with rapamycin reverts some of the effects of CCM loss in the latter but without affecting the mRNA overexpression of *Klf2* or *Klf4*, making the immediate targets of the drug unclear [14].

In agreement with in vitro effects, treatment with rapamycin in vivo inhibits cavernoma development in several preclinical models [14, 25]. However, the effects of rapamycin are not clear when a chronic model of CCM development and lower doses of the drug are used [26].

In this study, we investigated the effects of propranolol and rapamycin on *Ccm3* KO mBMEC and evaluated rapamycin‐based combinations in acute and chronic in vivo models of cavernomas. While neither treatment reversed known effects of CCM deficiency on protein membrane distribution, our unbiased transcriptomic analysis of drug effects revealed that rapamycin partially reverses the expression of genes dysregulated by *Ccm3* loss. In contrast, propranolol failed to demonstrate any significant effect in reversing the *Ccm3*‐deficient phenotype, even under unbiased RNA‐seq analysis. This observation suggests that if propranolol exhibits therapeutic effects in CCMs, it is unlikely to be through directly reversing the CCM phenotype in endothelial cells. We also present novel evidence that rapamycin, in combination with the tyrosine kinase inhibitor lapatinib, significantly reduces lesion volume in a chronic model of cavernomas. This finding underscores the potential of drug combinations as a promising therapeutic strategy for cavernomas, warranting further investigation.

## 2 Materials and Methods

### 2.1 Mouse Experiments

All experiments involving animals were performed following the ARRIVE guidelines [27]. The number of mice per experimental group is specified in the figure legends.

All mice used in this study, both for the isolation of brain endothelial cells and for in vivo treatments, were maintained in a C57BL/6J background from those developed by the Elisabetta Dejana laboratory, crossing *Pdcd10* ^fl/fl^ mice [8] with *cdh5* *(PAC)‐CreERT2* mice [28], as described in [8].

Male and female mice were used in the experiments, as no sex differences have been reported in the development of cavernomas.

For acute cavernoma induction (acute model), pups were injected at postnatal day 1 (P1) intragastrically with 50 μg tamoxifen (Merck Life Sciences #T5648) to obtain *Ccm3* ^iEC/iEC^ mice and induce CCM lesions. To induce the chronic cavernoma lesions (chronic model), pups were injected at P2 intragastrically with 2.5 μg tamoxifen to obtain *Ccm3* ^iEC/iEC^ mice.

Treatment for each individual animal within a litter was randomly assigned at birth, independently of sex. The sample size was based on previously published reports on cavernoma treatments using the same animal model.

Rapamycin (MedChemExpress #HY‐102129) was dissolved in DMSO and injected intraperitoneally at a dose of 1.5 mg/Kg. Lapatinib (MedChemExpress #HY‐50898) was dissolved in DMSO and then diluted in PBS, either alone or in combination with rapamycin, and injected intraperitoneally at a dose of 300 mg/kg. Control animals were administered a volume of DMSO equal to that received by treated mice.

In the acute model, animals were injected intraperitoneally with rapamycin, lapatinib, a combination of both drugs, or vehicle from postnatal day 2 (P2) to P6 and euthanised at P8 by CO_2_ inhalation followed by cervical dislocation. For the chronic model, adult mice were injected intraperitoneally with rapamycin, lapatinib, rapamycin + lapatinib, or vehicle from P30 to P39, following the same dosage regime and euthanised at P40 by CO_2_ inhalation followed by cervical dislocation.

Brains were collected and fixed with formalin until staining with Lugol's iodine (Merck Life Sci, #L6146) at 50% in water solution for 96 h (acute model) or 5 days (chronic model) [29]. Micro‐CT imaging was conducted using a Skyscan 1272 system (Bruker, Kontich, Belgium) at 100 kV and 100 μA, with a 10 μm pixel size, 1,150 ms exposure time, and a 0.4° rotation step, using an Al 0.5 + Cu 0.038 filter. Image projections were reconstructed with NRecon software (Bruker) and rendered using CTVox (Bruker). Volumetric segmentation and quantification of the total volume of CCM lesions in each animal were carried out using CTAn software (Bruker). All imaging and volume quantification were performed in a blind manner, with reconstruction parameters kept constant throughout the analysis. Those litters in which the mean cavernoma volume of untreated animals was less than 0.05 mm^3^ in acute induction or 0.5 mm^3^ in chronic induction were excluded.

### 2.2 Isolation of mBMEC and Treatments

mBMEC were isolated from adult 8–10‐week *Cdh5* (PAC)‐CreERT2/*Pdcd10* ^fl/fl^ mice. Mice were euthanised, and brain tissue was harvested, and tissue homogenisation and digestion were performed as described by Czupalla [30]. Then, the cells were resuspended and cultured as described by Assmann with slight modifications [31]. After cell isolation, cells were seeded onto plates coated with collagen I (Merck Life Sciences #C3867) in DMEM supplemented with 15% (v/v) fetal bovine serum (Gibco #10270–106), 1% v/v Penicillin/Streptomycin/L‐Glutamine (Corning 30–009‐CI), 100 μg/mL heparin (Merck Life Sci #H3149), and 50 μg/mL endothelial cell growth supplement (ECGS; Merck Life Sci #E2759). Cells were maintained at 37°C in 95% air and 5% CO_2_.

After 24 h, endothelial cells were selected using 4 μg/mL puromycin for 3 days, after which Cre recombinase activity was induced using 5 μg/mL 4‐hydroxy‐tamoxifen (Merck Life Sciences #SMC1666).

Passage 2 mBMEC were treated with either propranolol (Merck Life Sci #P0884), rapamycin (HY‐102129), lapatinib (MedChemExpress #HY‐50898), or lapatinib + rapamycin at the concentrations and time points indicated in the corresponding figure legends.

### 2.3 Immunofluorescence and Quantification

Passage 2 mBMEC were grown on coverslips pretreated with collagen I, allowed to reach confluency and treated for the times indicated in the corresponding figure legends. Then, coverslips were fixed using 4% paraformaldehyde in PBS pH 7.4, permeabilised with Triton X‐100 0.5% in PBS and processed for immunofluorescence using 10% FBS as blocking solution for 60 min. Primary antibodies used for immunostaining are listed in Table S1. Fluorescence‐conjugated Alexa Fluor 488 secondary antibodies (1:250, Invitrogen #A11001 or #A11008) were used and counterstained with DAPI (1:500). Coverslips were mounted with Fluoroshield (Merck Life Sci #F6182).

Images were acquired using a Leica DM4‐B and Leica K5 camera microscope or a Leica confocal microscope equipped with an HCX PL APO CS 63×/1.32 objective. Images were processed with the LAS X (Leica) and quantified using ImageJ (National Institutes of Health) software. The junctional/total ratio of VE‐cadherin and β‐catenin was calculated according to Colás‐Algora et al. [32]. Briefly, an area containing the whole cell was created and then made 10 pixels smaller to exclude the junctional region. This image was then subtracted from the original region to generate a region representing the fluorescence intensity at the cell periphery. The raw pixel intensity surface VE‐cadherin was divided by the corresponding area to yield a parameter that could be compared across the different images.

### 2.4 Protein Immunoblotting

Western blotting was performed by standard procedures after preparation of mBMEC extracts in cold radioimmunoprecipitation assay (RIPA) lysis buffer plus protease and phosphatase inhibitors. For KLF2 detection, the lysis buffer was 80 mM Tris–HCl, pH 7.8; 10% glycerol; 2% SDS, 1% β‐mercaptoethanol, plus protease and phosphatase inhibitors. Blots were developed using ECL Chemiluminescent detection reagents (Thermo Fisher Scientific Inc., Waltham, MA, USA #32106) and ChemiDocTM MP (Biograd). The software ImageJ (version 1.52 s, National Institute of Health, Bethesda, MD, USA) was used to quantify the Western blot signals. Primary antibodies used for Western blot are listed in Table S1. The secondary HRP‐conjugated antibodies used were 1 mg/mL Invitrogen #A16072 and #A16035.

### 2.5 RNA Extraction, Sequencing and Data Analysis

Total RNAs were extracted using the Trizol reagent (Life Technologies), according to the manufacturer's protocol. For RNA‐seq, RNA integrity and quantity of the samples were analysed using a NanoDrop spectrophotometer, followed by agarose gel validation.

For RT‐qPCR, RNA was reverse‐transcribed using random hexamers, and the mRNA expression level was determined using PowerUp SYBR Green Master Mix (Thermo Fisher Scientific Inc., Waltham, MA, USA #A25778) using a StepOnePlus Real‐Time PCR System. *Gapdh* mRNA levels were used as an internal control, and the 2‐^ΔΔCT^ method was used for analysis of the data. Each control value (*Ccm3* WT) was normalised to 1, and *Ccm3* KO values were relative to the control.

Oligonucleotides used for qPCR are listed in Table S2.

### 2.6 Bioinformatic Analysis of RNA‐Seq

RNA was sent to Novogene for RNA sequencing. Libraries were prepared using Illumina's mRNA‐Seq Poly(A) selection protocol and sequenced on a NovaSeq platform (paired‐end 150 bp, unstranded) with a depth exceeding 20 million reads per sample. Initial quality filtering was performed by Novogene prior to delivery of clean reads. Raw reads were filtered according to the following criteria: Removal of reads containing adapter sequences, removal of reads in which ambiguous nucleotides (N) constituted more than 10% of either read, and removal of reads in which more than 50% of bases had a Phred quality score ≤ 5.

The strandedness of the libraries was verified using *how_are_we_stranded_here*, and read quality was assessed with *FastQC* (v0.12.1). Samples were aligned to the Genome Reference Consortium Mouse Build 39 (GRCm39) with Ensembl annotation release 104 using *STAR* (v2.7.9a). Gene quantification was computed using *HTSeq* (v.0.13.5), and normalisation was performed using *DESeq2* (v.1.34.0) in *R* (v.4.2.2). Differentially expressed genes were identified using an adjusted q‐value < 0.05 and absolute fold change > 1.5 (Benjamini–Hochberg correction). All heatmaps, principal component analyses and volcano plots were produced in *R* (v4.1.2) using *tidyverse* (v2.0.0), *dplyr* (v1.1.4), *tibble* (v3.2.1), *ComplexHeatmap* (v2.10.0), *ggplot2* (v4.0.0) and *ggrepel* (v0.9.3), then refined and exported using *circlise* (v0.4.16), *viridisLite* (v0.4.2) and *svglite* (v2.1.3).

Gene Ontology (GO) analyses were conducted using the **DAVID** functional‐annotation platform (Database for Annotation, Visualisation, and Integrated Discovery; https://david.ncifcrf.gov/tools.jsp), as described.

All analyses were executed within a reproducible environment managed through *Conda* (v4.8.3) to ensure version control and computational reproducibility and performed within *RStudio* (Build 467).

Pre‐ranked Gene Set Enrichment Analysis (GSEA) [33] was performed on genes ranked by DESeq2 using the mouse MH: Hallmark gene sets MSigDB collection [34].

### 2.7 Statistics

Statistical analysis was performed using GraphPad software (https://www.graphpad.com/), version 7.0, San Diego, CA, applying the two‐sample, two‐tailed Student's test when 2 groups were compared, and two‐way ANOVA analysis with a Tukey's multiple comparison test when comparing > 2 groups. For cavernoma volumes, Welch's ANOVA test followed by a Dunnett's T3 multiple comparisons test was used, based on unequal variances of the different groups. A value of *p* < 0.05 was considered significant. All graphs represent the mean ± SEM with dots indicating individual values.

## 3 Results

To investigate the effects of drugs proposed for cavernoma treatment, we used mouse brain microvascular endothelial cells (mBMEC) derived from mice carrying a floxed *Ccm3* allele [35] and a tamoxifen‐inducible cre recombinase (*cdh5*‐creERT2) [28]. Treatment of cultures with 4‐OH‐tamoxifen (4‐OHT) for 4 days [36] markedly reduced levels of *Ccm3* mRNA and protein compared to untreated cells (Figure S1A,B). These *Ccm3*‐deficient cells recapitulated hallmark CCM phenotypes, including elevated *Klf2* and *Klf4* mRNA levels (Figure S1C) and reduced junctional levels of β‐catenin and VE‐cadherin (Figure S1D). Also, *Ccm3* inactivation altered the expression of 168 genes as seen in RNAseq analysis (Figure S2A and Table S3). Analysis of the differentially expressed genes (Figure S2B) showed enrichment in biological processes that have been reported to be related to cavernomas, such as angiogenesis, cell adhesion, cell migration or extracellular matrix organisation [37], which confirms that mBMECs are a good model to study the effects of CCM deficiency.

**FIGURE 1:**
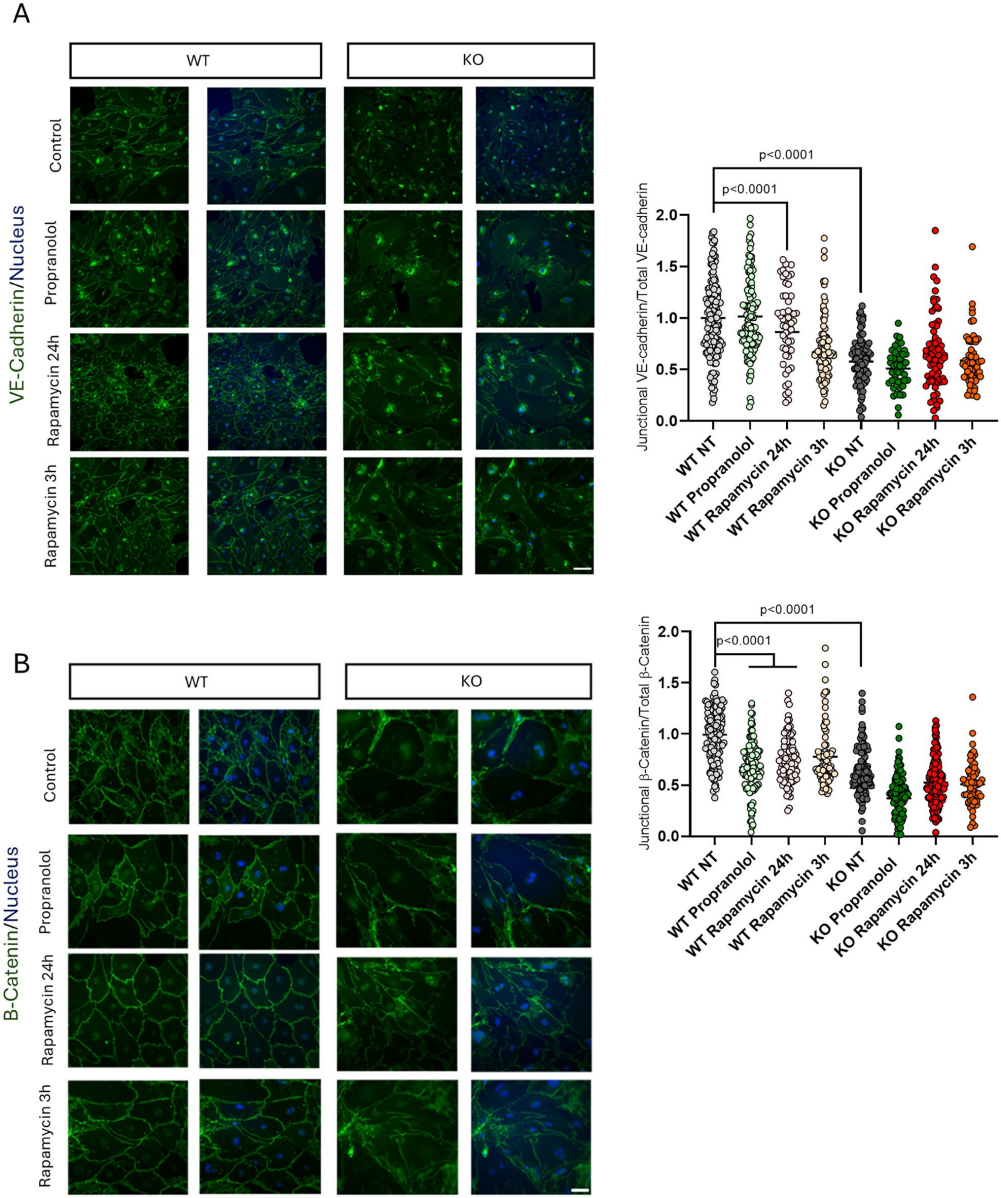
Rapamycin or propranolol do not recover the abnormal distribution of VE‐cadherin or β‐catenin in *Ccm3* KO mBMEC. (A) Distribution of VE‐cadherin. WT or KO mBMEC were either left untreated or treated with 10 μM propranolol for 24 h, 100 nM rapamycin for 24 h, or 100 nM rapamycin for 3 h, and immunofluorescence was performed for VE‐cadherin, counterstained with DAPI. Upper panels: Representative photographs of the immunofluorescence with staining for VE‐cadherin (green) and DNA (blue). Scale bar is 50 μm. The graph shows the ratio of junctional/total β‐catenin of individual cells after each treatment of > 100 cells per treatment from 4 independent biological replicates (cell cultures), referenced to the average of untreated WT mBMEC of the same cell culture. Each cell culture was obtained from a pool of 3 to 4 brains. *p*‐values are from ANOVA analysis with a Tukey's multiple comparison test. (B) Distribution of β‐catenin in WT and KO mBMEC. Upper panels: Representative photographs of the immunofluorescence with staining for β‐catenin (green) and DNA (blue). Scale bar is 50 μm. The graph shows the ratio of junctional/total β‐catenin of individual cells after each treatment of > 100 cells per treatment from 4 independent biological replicates (cell cultures), referenced to the average of untreated WT mBMEC of the same cell culture. Each cell culture was obtained from a pool of 3 to 4 brains. *p*‐values are from a two‐way ANOVA analysis with a Tukey's multiple comparison test.

We sought to elucidate the specific effects of propranolol and rapamycin within this defined cellular model of CCM. We treated cells for 24 h with 10 μM propranolol, based on its plasma levels in treated hypertensive patients and the distribution of the drug in brain tissue [38, 39]. For rapamycin, we treated cells for 3 h or 24 h at a 100 nM concentration, based on the peak and trough levels attained in plasma after mice dosing [40, 41, 42]. First, we analysed the localisation of VE‐cadherin and β‐catenin. VE‐cadherin or β‐catenin distribution to the cell junctions was not affected by treatments in *Ccm3* KO cells (Figure 1A), although, surprisingly, rapamycin reduced the amount of both proteins and propranolol the amount of β‐catenin in wild‐type cells after a 24‐h exposure (Figure 1B). Thus, neither rapamycin nor propranolol restored the abnormal distribution of VE‐cadherin or β‐catenin in *Ccm3* KO mBMEC.

**FIGURE 2:**
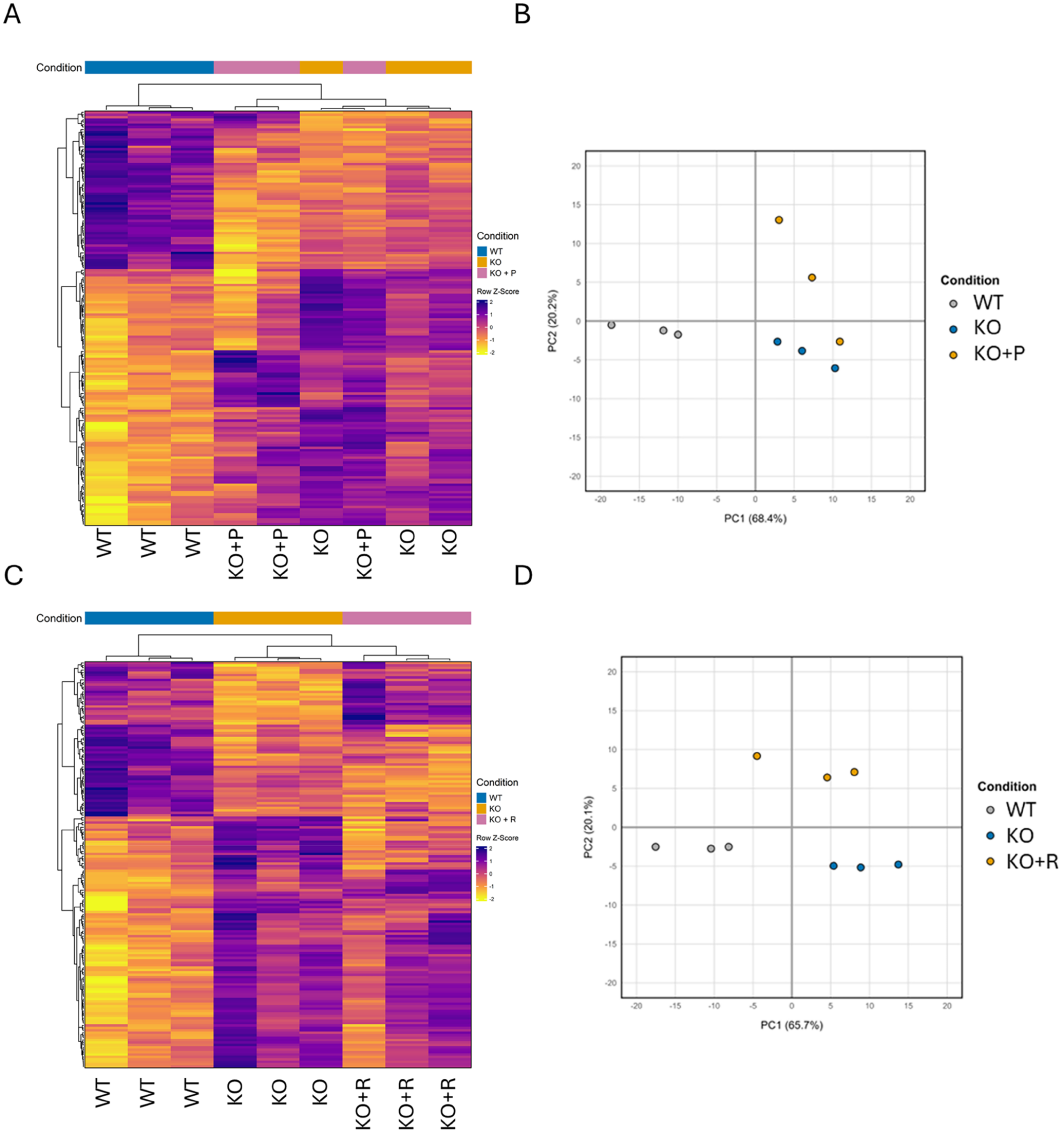
Rapamycin, but not propranolol, can partially revert the transcriptional effect of *Ccm3* inactivation in mBMEC. WT and KO mBMEC were left untreated or treated with 10 μM propranolol for 24 h (KO+*P*) or 100 nM rapamycin for 24 h (KO+*R*). RNAseq analysis was performed, and their expression profile was compared using genes differentially expressed in KO vs. WT mBMEC. Three independent biological replicates (cell cultures) were analysed in all cases. Each cell culture was obtained from a pool of 3 to 4 brains. (A) Heatmap analysis of WT, KO, and KO+*P*, showing that KO+*P* cells cluster with KO cells. (B) Principal component analysis of WT, KO, and KO+*P* mBMEC, showing a displacement of KO vs. WT mBMEC in the horizontal axis that is not reversed by treatment with propranolol. (C) Heatmap analysis of WT, KO, and KO+*R*, showing that treated KO+*R* mBMEC no longer cluster with KO mBMEC. (D) Principal component analysis of WT, KO, and KO+*R* mBMEC, showing a displacement of KO vs. WT mBMEC in the horizontal axis that is partially reverted by treatment with rapamycin.

To study the effect of both treatments in an unbiased manner, we performed an RNA‐seq analysis of *Ccm3* KO cells after 24 h treatments and analysed their effect on the 168 genes that were differentially expressed in KO vs. WT cells, which reflected the effect of *Ccm3* loss on brain endothelial cells. Treatment with propranolol affected the expression of several of these genes and reversed more than 1.5‐fold the effect of the lack of *Ccm3* on the expression of 19 of them (Figure 2A and Table S3). However, when a principal component analysis (PCA) was performed comparing WT, untreated and treated KO cells, the effect of propranolol was shown not to reverse the effects of *Ccm3* deficiency but shifted samples along a principal component orthogonal to the *Ccm3* deficiency axis, without reversing it (Figure 2B). In contrast, treatment with rapamycin could reverse more than 1.5‐fold the expression of 79 of the 168 genes deregulated in CCM3 KO cells, and partially recovered the displacement induced by the loss of *Ccm3*, suggesting a normalisation of gene expression and thus a possible therapeutic effect in these cells (Figure 2D and Table S3). This is also reflected in the heatmap of the DEGs, where KO cells treated with rapamycin cluster apart from untreated KO cells (Figure 2C). To see whether rapamycin has effects other than reversion of the CCM phenotype in mBMEC, we also performed a GSEA (Gene Set Enrichment Assay) of genes affected by rapamycin treatment in both *Ccm3* WT and KO cells using the MSigDB Hallmark collection (Figure S3). No gene set was upregulated by rapamycin treatment in *Ccm3* WT or KO cells, while mTORC1 signalling was downregulated in both, in agreement with the known effect of the drug. Rapamycin also downregulated several gene sets related to cell proliferation in *Ccm3* KO cells but not in WT cells, where the effect did not reach statistical significance, consistent with its well‐known effect on cell proliferation.

**FIGURE 3:**
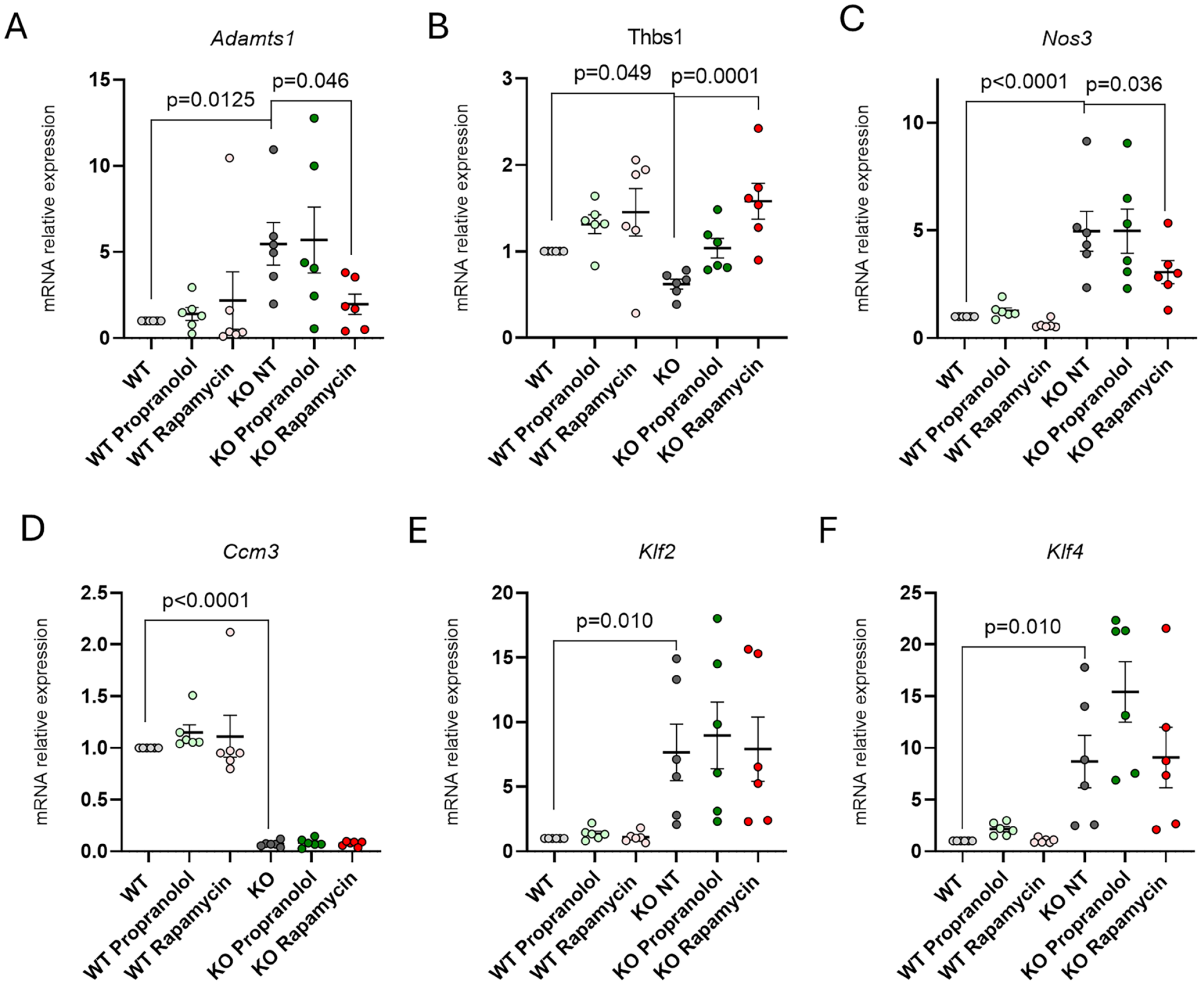
Rapamycin, but not propranolol, reverts the expression of genes dependent on KLF2/4, without affecting *Klf2* or *Klf4* mRNA expression. WT or KO mBMEC were either left untreated or treated with 10 μM propranolol for 24 h or 100 nM rapamycin for 24 h. RNA was extracted and RT‐qPCR was performed using GAPDH as a reference gene. Each graph shows the average and SEM of mRNA relative expression and the individual values of six independent biological replicates (cell cultures), referenced to the levels of untreated WT mBMEC of the same cell culture. Each cell culture was obtained from a pool of 3 to 4 brains. *p*‐values are from a two‐way ANOVA analysis with a Tukey's multiple comparison test. (A) Expression of *Adamts1*, (B) Expression of *Thbs1*, (C) Expression of *Nos3*, (D) Expression of *Ccm3*, (E) Expression of *Klf2*, and (F) Expression of *Klf4*.

We next wanted to further analyse the partial recovery of the CCM transcriptional phenotype by rapamycin. In‐depth analysis of the genes recovered by rapamycin showed that they included some of the genes known to be dependent on KLF2/4 transcription factors, which are deregulated in CCM‐deficient cells. This included genes such as *Adamts1*, overexpressed in CCM‐deficient cells [43, 44], or *Thbs1*, downregulated in CCM‐deficient cells [37]. To confirm this effect, we performed RT‐qPCRs in cell preparations different from those used for RNA‐seq. *Adamts1* was confirmed as overexpressed in *Ccm3* KO mBMEC and reduced by rapamycin (Figure 3A), while *Thbs1* was downregulated in KO cells, with rapamycin recovering it (Figure 3B). *Nos3*, a gene regulated by KLF2/4 and important in CCM pathogenesis [45], was also overexpressed in KO cells and reduced by rapamycin when checked by RT‐qPCR (Figure 3C). On the contrary, *Ccm3*, *Klf2* and *Klf4* were not affected by rapamycin treatment in *Ccm3* KO cells (Figure 3D–F), which is consistent with its described effects in other CCM‐deficient endothelial cells [14]. Contrary to the effects of rapamycin, and in accordance with the RNA‐seq results, propranolol did not influence the expression of any of these genes.

**FIGURE 4:**
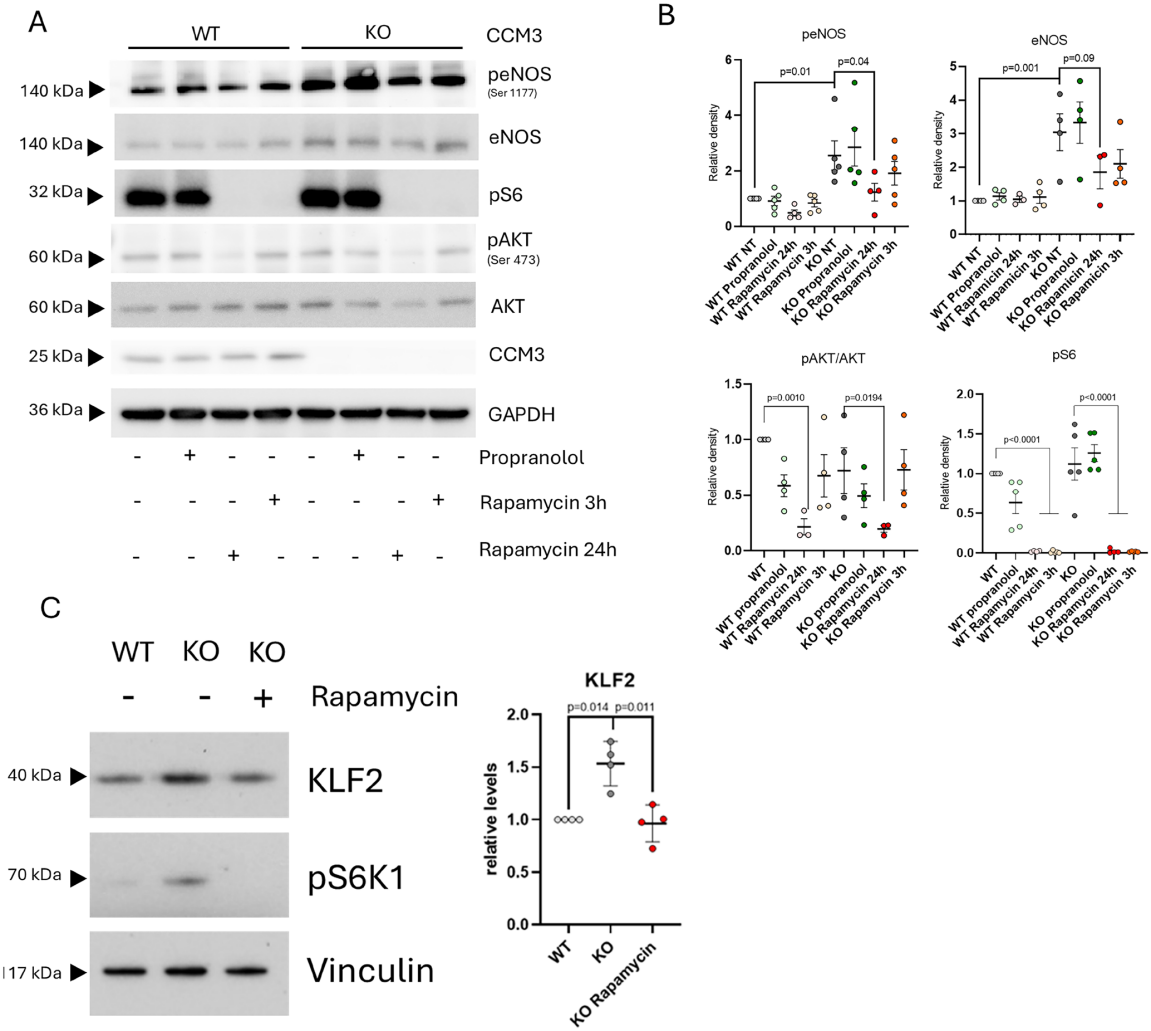
Effect of rapamycin on eNOS and KLF2 protein levels. (A) Western blot of mBMEC proteins after treatments. Extracts were prepared from WT and KO mBMEC treated with 100 nM rapamycin for 3 or 24 h, or with 10 μM propranolol for 24 h, and western blots were performed for total eNOS, eNOS phosphorylated in Ser 1177 (peNOS), phosphorylated S6 (pS6), AKT phosphorylated in Ser 473 (pAKT), total AKT, CCM3, with GAPDH as a loading control. (B) Average and SEM of the intensity of peNOS, eNOS, pAKT/AKT ratio and pS6 from four independent biological replicates (cell cultures), referenced to the average of untreated WT mBMEC of the same cell culture. Each cell culture was obtained from a pool of 3 to 4 brains. *p*‐values are from ANOVA analysis with a Tukey's multiple comparison test. (C) Rapamycin diminishes KLF2 protein levels in *Ccm3*‐deficient cells. KO and WT mBMEC were left untreated or treated with 100 nM rapamycin for 24 h. Western blots were performed for KLF2, vinculin and pS6K1. Left panel, representative western blot. Right graph, average and SEM of corrected intensity of KLF2 of western blots from four biological replicates (cell cultures), referenced to the average of untreated WT mBMEC of the same cell culture. Each cell culture was obtained from a pool of 3 to 4 brains. *p*‐values are from a two‐way ANOVA analysis with a Tukey's multiple comparison test.

Overexpression of *Nos3* is important for lesion development through a feedback loop involving NO production by cavernoma endothelial cells and the ensuing secretion of VEGF by astrocytes that contributes to lesion enlargement [45]. Thus, we wanted to see if the effect of rapamycin on *Nos3* mRNA was reflected at the protein level. As seen in Figure 4A,B, both eNOS protein levels and its phosphorylation in serine 1177 were stimulated in *Ccm3*‐deficient cells, and this was reversed when they were treated with rapamycin for 24 h. The same western blot shows that *Ccm3*‐deficient cells showed a trend toward higher S6 phosphorylation with no change in Akt phosphorylation in S473, which is in agreement with the effects of *Ccm1* KO in brain endothelial cells and HuVECs, as reported by Ren et al. [14]. Rapamycin acted as expected both in WT and KO cells, inhibiting S6 phosphorylation after 3 and 24 h of treatment and diminishing AKT phosphorylation in S473 only after 24 h.

The effect of rapamycin on known transcriptional targets of KLF2 and KLF4, together with its lack of effect on the expression of these transcription factors at the mRNA level, hints at a possible post‐transcriptional effect of the drug on *Klf2* and/or *Klf4*, probably through the known effects of rapamycin on translation. However, there is no apparent effect on *Klf4*, as its protein levels have been shown not to change with rapamycin in *Ccm1*‐deficient endothelial cells, both in vivo and in vitro [14]. Intriguingly, in a different setting, Wang et al. have shown that rapamycin inhibits *Klf2* by post‐transcriptional mechanisms in endothelial cells [46]. When we checked KLF2 protein levels in *Ccm3* KO cells, we saw that rapamycin treatment could diminish KLF2 protein (Figure 4C), even if it did not inhibit its mRNA. These data indicate that rapamycin reduces KLF2 protein independently of its mRNA level, suggesting a post‐transcriptional mechanism underlying its effect on KLF2/4 target genes.

**FIGURE 5:**
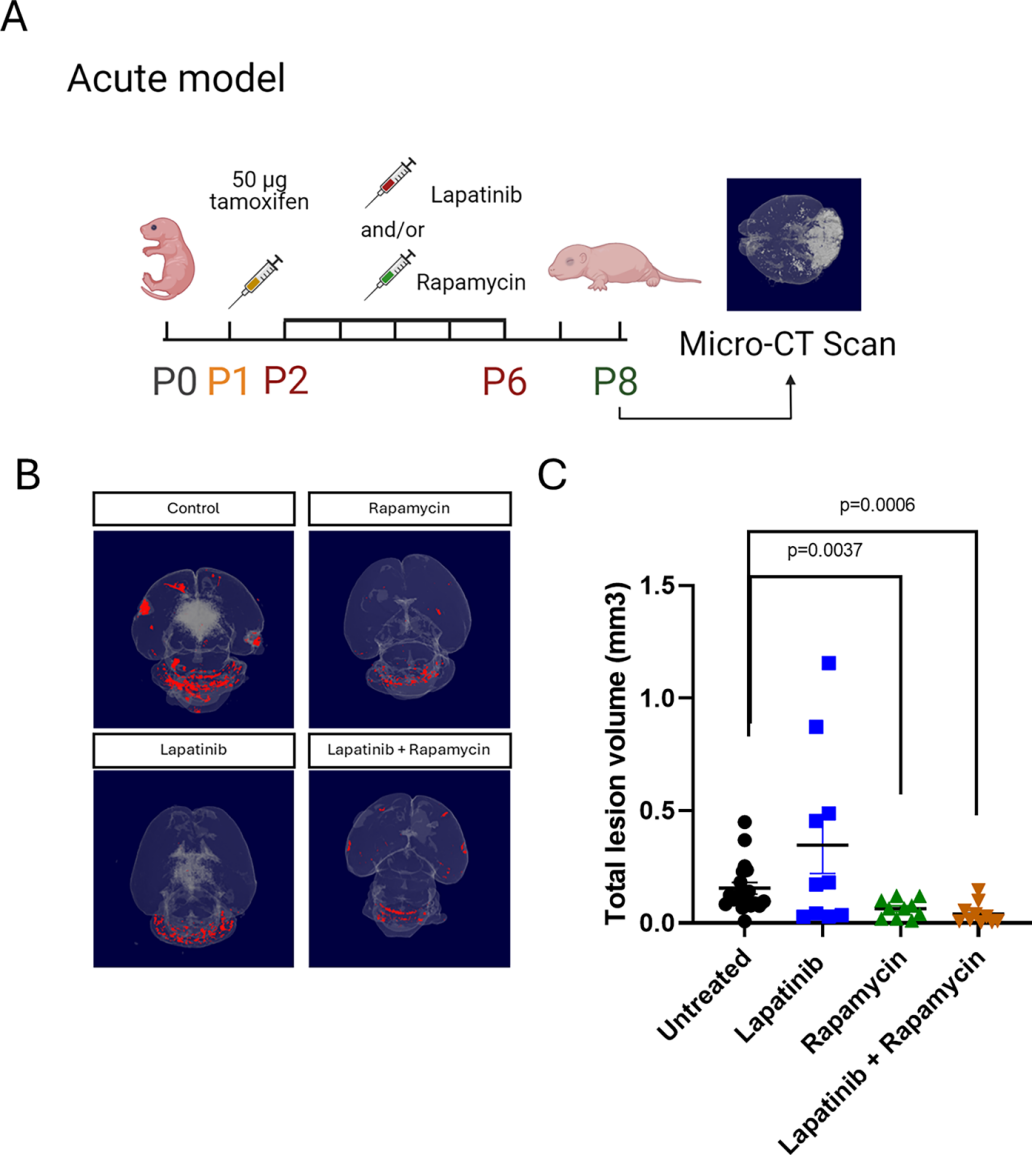
Rapamycin, but not lapatinib, can inhibit acute cavernoma development. (A) Scheme showing the induction and treatment of cavernomas. At P1, 50 μg tamoxifen was administered to newborn Cdh5 (PAC)‐CreERT2/*Pdcd10* ^fl/fl^ mice. Rapamycin and/or lapatinib were injected at days P2 to P6, and mice were euthanised at P8, brains extracted and analysed by micro‐CT. (B) Micro‐CT images of the brains of P8 mice with induced cavernomas, either untreated or treated with lapatinib, rapamycin, or lapatinib + rapamycin. (C) Volume of cavernomas in untreated mice (*n* = 19 mice), or mice treated with lapatinib (*n* = 10 mice), rapamycin (*n* = 10 mice), or lapatinib + rapamycin (*n* = 10 mice). Total volumes of cavernomas per brain were calculated from micro‐CT images. *p*‐values are from Welch's ANOVA test followed by a Dunnett's T3 multiple comparisons test. Panel A was created with biorender.com.

The effects of rapamycin in the partial reversal of alterations induced by the lack of *Ccm3* reinforce the idea that it could be used for the treatment of both growing and mature cavernoma lesions, either alone or in combination with other agents. Rapamycin has been shown to prevent the development of cavernomas using animal models of the disease, both in lesions with only mutations in a CCM gene and in those harbouring also *Pik3ca* mutations [14]. However, concerns have been raised about these results based on the high doses of rapamycin used, which may have achieved supratherapeutic levels that cannot be safely tolerated in humans [42]. The trough levels of rapamycin that are usually targeted in plasma for benign conditions are 5–15 ng/mL [42, 47]. Thus, we decided to induce cavernoma in neonatal *Cdh5* (PAC)‐CreERT2/*Pdcd10* ^fl/fl^ mice [28] and administer 1.5 mg/Kg rapamycin intraperitoneally, a lower dose than the 2 mg/Kg used by Bishu et al. to attain trough levels of 13.8 ng/mL [47] (Figure 5A). Under this regime, rapamycin attenuated cavernoma development but did not fully prevent lesion formation, as happens when higher doses are used [14, 25, 48] (Figure 5B,C).

We then considered the possibility of using rapamycin in combination with other agents for the treatment of cavernomas. Since propranolol had no apparent effect in our cellular system, we turned to the HER2/EGFR inhibitor lapatinib, as we had previously shown that *Ccm3*‐deficient endothelial cells have higher levels of EGF receptor and are more susceptible to apoptosis after its inhibition with lapatinib in vitro [13]. Further, it is known that high levels of PI3K activity in tumours make them resistant to EGF receptor inhibition, and rapamycin enhances the effect of EGFR inhibition in breast cancer cells [49].

To address this point, we examined the effects of lapatinib on gene expression in Ccm3‐deficient endothelial cells as compared to rapamycin. Lapatinib, either alone or in combination with rapamycin, had no apparent effect on the mRNA levels of *Klf2* and, contrary to rapamycin, it did not reverse the expression of the Klf2/4‐dependent genes *Adamts1* or *Thbs1* (Figure S4A–C). We have previously shown that lapatinib induces apoptosis in *Ccm3*‐deficient endothelial cells, and it has also been reported to upregulate pro‐apoptotic genes in HER2+ cancer cells, including *Bcl2l11* (also known as *Bim*), *Tnfrsf10b*, and *Bax* [50, 51, 52]. In our system, lapatinib induced *Tnfrsf10b* expression (Figure S4D) and *Bax* when combined with rapamycin (Figure S4E), while it had no apparent effect on *Bcl2l11* expression (Figure S4F).

Together, these results indicate that lapatinib exerts effects distinct from and potentially complementary to those of rapamycin in Ccm3‐deficient endothelial cells, supporting the evaluation of their combined use in vivo.

Treatment of *Cdh5* (PAC)‐CreERT2/*Pdcd10* ^fl/fl^ mice with cavernomas induced neonatally with 300 mg/Kg lapatinib alone did not significantly prevent cavernoma development. However, the combined rapamycin/lapatinib treatment resulted in a trend toward lower volume of the cerebellum occupied with lesions as compared with the already low volume of cavernomas in mice treated with rapamycin alone (Figure 5B,C). This suggested that combined inhibition of mTORC1 and the EGF receptor family could be a useful strategy to limit cavernoma burden, and that it could have a greater effect in situations where rapamycin alone is less effective, such as cavernomas that have already developed.

**FIGURE 6:**
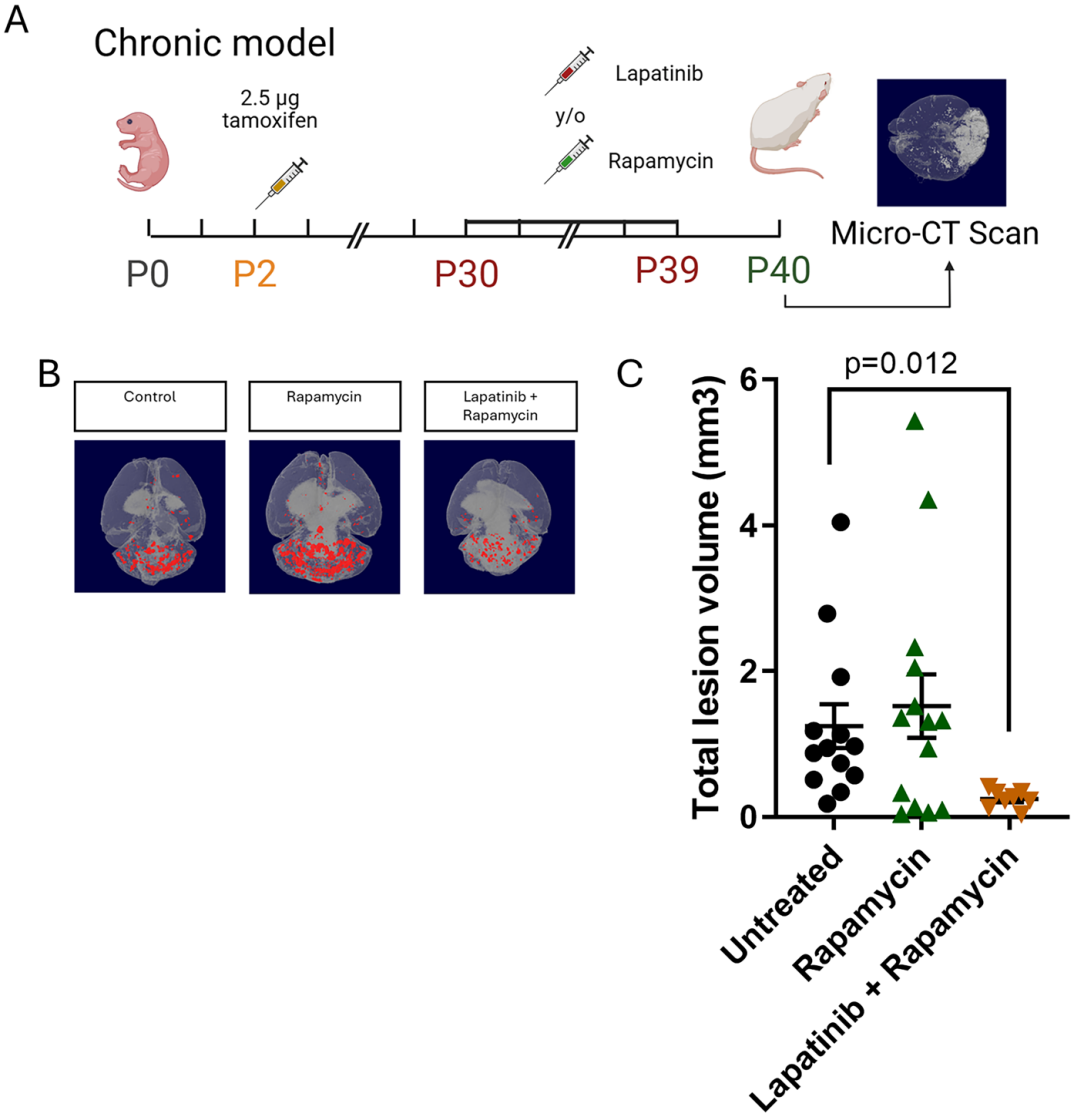
Rapamycin/lapatinib combination can diminish cavernoma volume when used after cavernoma development. (A) Scheme showing the induction and treatment of cavernomas. At P2, 2.5 μg tamoxifen was administered to Cdh5 (PAC)‐CreERT2/*Pdcd10* ^fl/fl^ mice. Rapamycin or rapamycin + lapatinib were injected at days P30 to P39, and mice were euthanised at P40, brains extracted and analysed by micro‐CT. (B) Micro‐CT images of the brains of P30 mice with induced cavernomas, either untreated or treated with rapamycin or lapatinib + rapamycin. (C) Volume of cavernomas in untreated mice (*n* = 13 mice), or mice treated with rapamycin (*n* = 14 mice) or lapatinib + rapamycin (*n* = 8 mice). Total volumes of cavernomas per brain were calculated from micro‐CT images. *p*‐values are from Welch's ANOVA test followed by a Dunnett's T3 multiple comparisons test. Panel A was created with biorender.com.

Being able to inhibit lesions that have already developed would be an optimal strategy for the treatment of cavernomas. Unfortunately, treatment of chronic lesions with pharmacologically relevant doses of rapamycin does not result in lesion improvement [26]. We wanted to see if the addition of lapatinib could help reduce the lesion volume of more mature lesions. We used a model of chronic cavernoma developed for the same animals used for acute cavernoma development by Malinverno et al. [53], and treated them with an injection of rapamycin alone or in combination with lapatinib every 24 h from postnatal day 30 (P30) to P39, measuring cavernoma volume at P40 (Figure 6A). Consistent with recent reports [26], rapamycin alone did not affect the evolution of lesions in this model, whereas combined rapamycin and lapatinib treatment significantly reduced lesion volume (Figure 6B,C), suggesting that the combination of rapamycin with inhibition of tyrosine kinase receptors may be especially useful to treat established cavernomas.

## 4 Discussion

We analysed how rapamycin and propranolol influence the transcriptomic signature of *Ccm3* KO brain endothelial cells and assessed their ability to revert it toward the WT profile. Propranolol did not measurably correct the *Ccm3* KO transcriptional phenotype at the doses and treatment times used, whereas rapamycin partially restored a subset of deregulated genes.

Rapamycin significantly impacts the expression of genes known to be deregulated in CCM‐deficient endothelial cells, such as *Nos3*, *Adamts1* and *Thbs1*. The deregulated expression of these genes in KO cells is dependent on the transcription factors KLF2 and KLF4. We also show that rapamycin diminishes KLF2 protein, consistent with a potential mechanism for the restoration of expression of KLF2/4 transcriptional targets in *Ccm3* KO cells. The effect of rapamycin seems to be selective on KLF2 as, even though we have not been able to detect KLF4 consistently in our cell model, rapamycin is known not to affect KLF4 protein levels in CCM‐deficient endothelial cells [14]. Our results add to the known effects of rapamycin on CCM‐deficient endothelial cells, such as inhibition of endothelial‐mesenchymal transition and of ROS production [17] and on endothelial progenitors [25]. Interestingly, while rapamycin influences several pathways altered in cavernomas, it does not affect them all. Specifically, rapamycin does not revert the altered distribution of β‐catenin or VE‐cadherin in the membrane of *Ccm3* KO cells, suggesting that junctional abnormalities in β‐catenin and VE‐cadherin are driven by KLF2‐independent mechanisms.

Several genes regulated by rapamycin are important in cavernoma pathogenesis, among which *Nos3* overexpression and the ensuing overproduction of NO in CCM‐deficient endothelial cells are known to be important in cavernoma pathogenesis [45], whereas inhibition of *Thbs1* also favours cavernoma development [37]. This underscores the potential of rapamycin as a component of a pharmacological strategy to treat cavernomas, even in the absence of *PIK3CA* mutations. This potential may be strengthened by the well‐known antiproliferative effect of rapamycin, which is reflected in this work by its effect on proliferation‐related gene expression in *Ccm3* KO cells. However, more experiments are needed to ascertain if this antiproliferative effect is sustained in time, as in other cellular models, it diminishes after a prolonged treatment [54]. Another limitation of rapamycin treatment of cerebral cavernous malformations is the possible side effects of the drug in wild‐type cells. In this respect, the negative effect of rapamycin on junctional VE‐cadherin and β‐catenin should be taken as a note of caution. On the other hand, the lack of off‐target effects of rapamycin on the phenotype of *Ccm3* WT cells, as assessed by GSEA analysis, suggests that these possible side effects are not strong enough to be reflected in consistent transcriptomic changes.

We also provide evidence that rapamycin, when combined with a drug possessing a distinct mechanism of action such as lapatinib, can improve chronic cavernoma lesions. This suggests that the combination of rapamycin and lapatinib, but not rapamycin alone, may improve cavernomas after they have already formed. A common characteristic of all proposed cavernoma treatments that have failed in clinical trials to date is their efficacy in preventing cavernoma development, rather than in improving existing cavernomas. However, in clinical settings, cavernomas are typically already detectable upon diagnosis. The activity of the rapamycin/lapatinib combination in improving cavernomas after 30 days of development warrants further evaluation in refined preclinical models incorporating longitudinal MRI assessment before and after treatment.

In contrast to rapamycin, propranolol exhibits no direct endothelial transcriptomic rescue at a clinically plausible concentration in this model. These results differ from reports showing phenotypic effects of propranolol in CCM‐deficient endothelial cells [21, 23, 55]. However, in those experiments, the concentration of propranolol used was significantly higher (50 to 100 μM), substantially higher than concentrations achievable in patients. Propranolol may also have beneficial effects on cavernoma development that are mediated by its actions in non‐endothelial cells. β1‐adrenergic signalling in neural and glial cells can modulate neurovascular unit function, inflammation and vascular permeability; thus, astrocytes or neurons may contribute to propranolol's protective effects by altering cytokine or VEGF release, oxidative stress, or blood–brain barrier support in CCM lesions [45]. Consistently, propranolol has been reported to reduce VEGF levels in CCM patients [55], and inhibition of VEGF signalling diminishes lesion burden in animal models [56]. In addition, propranolol improves pericyte coverage and vascular sealing in *Ccm3*‐deficient murine lesions [23], suggesting that reinforcement of pericyte–endothelial interactions, rather than direct correction of endothelial transcriptional defects, may also underlie lesionstabilisation.

Besides the use of endothelial cells in culture and a mouse model of cavernoma pathogenesis, our study is limited by the lack of validation in human primary endothelial cells. While cavernoma biology appears to be largely conserved across species, differential drug responses cannot be ruled out. Future studies in human models will therefore be important to extend these findings.

## 5 Conclusion

Overall, these data identify rapamycin as an effective modulator of the *Ccm3*‐deficient endothelial phenotype and support rapamycin–lapatinib combination therapy as a promising approach for treating established CCM lesions.

## Author Contributions

**Luis Díaz‐Gómez:** methodology, investigation, writing – review and editing, visualization. **Cristina Riobello:** methodology, software, writing – review and editing. **José E. Martínez:** methodology, investigation, writing – review and editing, visualization. **Diego González‐Pérez:** methodology, investigation, writing – review and editing. **Aurora Gómez‐Durán:** methodology, visualization. **Miguel Fidalgo:** methodology, investigation, visualization, software, writing – review and editing, formal analysis. **David Martínez‐Delgado:** methodology, software, data curation, validation, writing – review and editing, formal analysis. **Miriam Sartages:** methodology, investigation, writing – review and editing. **Eva M. Esquinas‐Román:** methodology, software, writing – review and editing. **Mar García‐Colomer:** conceptualization, methodology, investigation, writing – review and editing, visualization. **Marta Varela‐Rey:** methodology, software, visualization, writing – review and editing. **Celia M. Pombo:** conceptualization, methodology, investigation, validation, visualization, supervision, project administration, funding acquisition, writing – original draft, writing – review and editing, formal analysis. **Juan Zalvide:** conceptualization, investigation, funding acquisition, writing – original draft, methodology, validation, visualization, writing – review and editing, project administration, supervision, formal analysis.

## Funding

This work was supported by the Spanish Agencia Estatal de investigación and by “ERDF A way of making Europe” (projects PID2021‐123365OB‐I00 to CMP and JZ, PID2020‐119486RB‐100 and PID2023‐152685OB‐I00 to M.V.‐R) and by the Consellería de Cultura, Educación e Ordenación Universitaria, Xunta de Galicia (ED431C 2023/10 to C‐M‐P. and J.Z). M. García‐Colomer was a predoctoral fellow from Xunta de Galicia. The fellowship to E.M.E.R is supported by PRE2021‐099788, and to C.R.S by MU‐21‐UP2021‐03071902373A, both funded by the Spanish Agencia Estatal de Investigación.

## Ethics Statement

All procedures involving animals were performed in accordance with the European Parliament and of the Council Directive 2010/63/EU and Spanish legislation (RD 53/2013) and were approved by the Ethics Committee on Animal Welfare of the University of Santiago de Compostela (reference 15,010/19/003).

## Conflicts of Interest

The authors declare no conflicts of interest.

## Supporting information


**Figure S1:** Validation of *Ccm3* inactivation in mBMEC. Expression of *Ccm3* mRNA and protein and *Klf2* and *Klf4* mRNAs, and redistribution of VE‐cadherin and β‐catenin in *Ccm3* KO mBMEC.
**Figure S2:** Volcano plot and Gene Ontology analysis of gene expression of Ccm3 WT vs. KO mBMEC.
**Figure S3:** GSEA analysis of the transcriptomic effect of rapamycin treatment on Ccm3 WT and Ccm3 KO mBMEC.
**Figure S4:** Effects of Lapatinib treatment on Klf2/4‐dependent and proapoptotic genes in Ccm3 KO cells.
**Table S1:** Primary antibodies used for immunofluorescence or western blots.
**Table S2:** Oligonucleotides used for qPCR.
**Table S3:** Ccm3 KO vs. WT differentially expressed genes. Effect of propranolol or Rapamycin treatment in Ccm3 KO cells.

## Data Availability

The RNA‐seq data is available in the NCBI GEO repository (accession GSE298723). RNA‐seq differential expression bioinformatic analysis and figure creation are at https://github.com/dvidmd/GSE298723_Differential_Gene_Expression_Analysis_Garcia_Colomer_et_al.
