# Supplementary material for: Rapamycin Partially Reverts Cavernoma Endothelial Cell Phenotype and, When Combined With Lapatinib, Ameliorates Chronic Lesions
Source: J Cell Mol Med. 2026 Jul 9;30(13):e71280. doi: 10.1111/jcmm.71280 (PMC13351306; doi:10.1111/jcmm.71280)
Supplement: Supplementary file 1 — Figure S1: Validation of Ccm3 inactivation in mBMEC. Expression of Ccm3 mRNA and protein and Klf2 and Klf4 mRNAs, and redistribution of VE‐cadherin and β‐catenin in Ccm3 KO mBMEC. Figure S2: Volcano plot and Gene Ontology analysis of gene expression of Ccm3 WT vs. KO mBMEC. Figure S3: GSEA analysis of the transcriptomic effect of rapamycin treatment on Ccm3 WT and Ccm3 KO mBMEC. Figure S4: Effects of Lapatinib treatment on Klf2/4‐dependent and proapoptotic genes in Ccm3 KO cells. Table S1: Primary antibodies used for immunofluorescence or western blots. Table S2: Oligonucleotides used for qPCR. Table S3: Ccm3 KO vs. WT differentially expressed genes. Effect of propranolol or Rapamycin treatment in Ccm3 KO cells. [file JCMM-30-e71280-s001.docx]

**Supporting information**

**Rapamycin can revert CCM phenotype in brain endothelial cells and when combined with lapatinib ameliorates chronic lesions**

Mar García-Colomer, José E Martínez, Luis Díaz, Miriam Sartages, Eva M. Esquinas-Román, Cristina Riobello, David Martínez-Delgado, Diego González-Pérez, Aurora Gómez-Durán, Miguel Fidalgo, Marta Varela-Rey, Celia M Pombo, Juan Zalvide

**
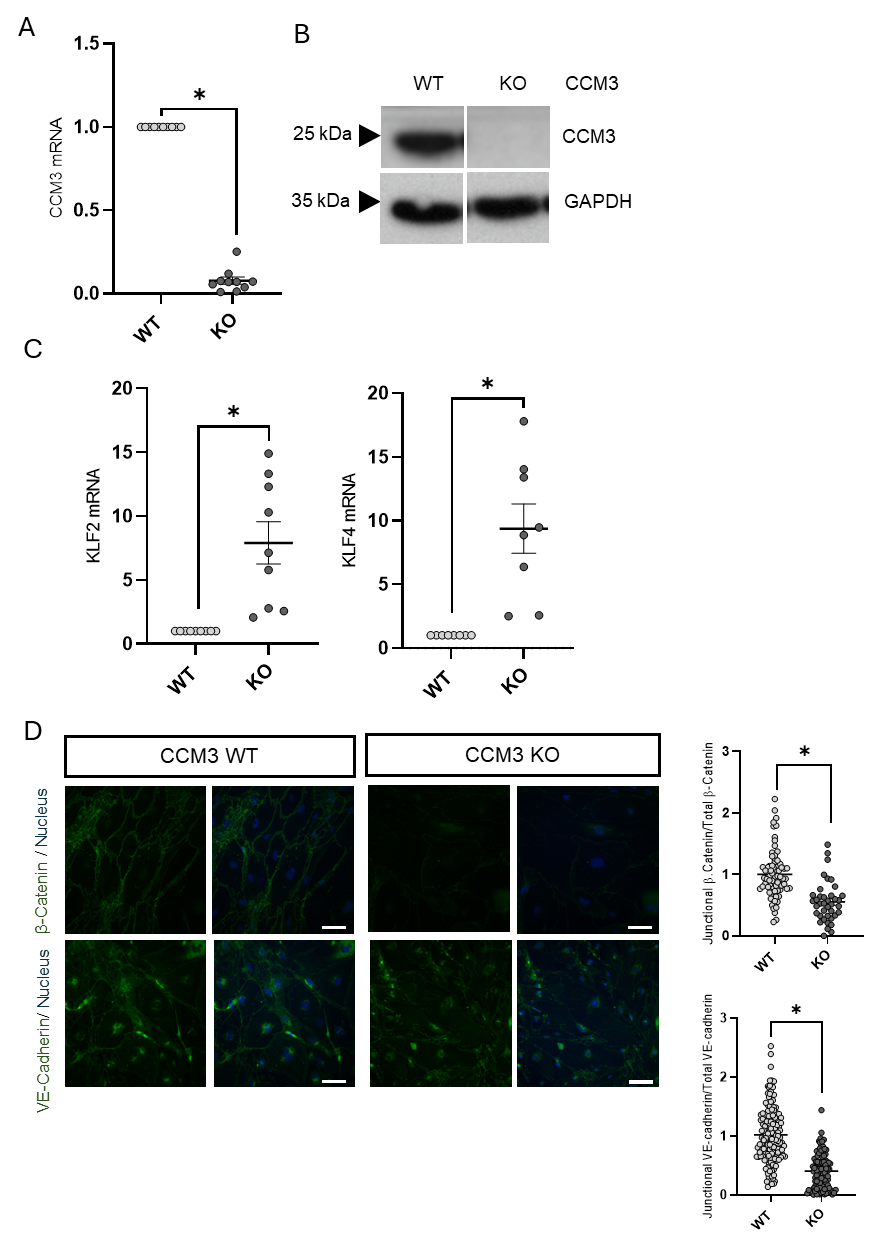
**

**Figure S1.** Loss of *Ccm3* in mBMEC induces *Klf2/4* upregulation and redistribution of β-catenin and VE-cadherin. mBMEC from PDCD10fl/fl cdh5(PAC)-CreERT2 mice were either treated with 4-hydroxytamoxifen to generate *Ccm3* KO cells (KO) or left untreated for *Ccm3* WT cells (WT). **A.** Downregulation of *Ccm3* mRNA in *Ccm3* KO mBMEC. An RT-qPCR was performed on *Ccm3* WT and KO cells of passage 2 and mRNA levels relative to GAPDH mRNA were normalized to those of *Ccm3* WT cells of each preparation. Graph shows the average and SEM of mRNA relative expression and the individual values of 10 independent biological replicates (cell cultures). Each cell culture was obtained from a pool of 3 to 4 brains. P-Value from two-sample, two-tailed Student’s t test. **B**. Downregulation of *Ccm3* in *Ccm3* KO mBMEC. Cells were treated as in A and western blots were performed in *Ccm3* WT and KO cells. Shown is a representative western blot. **C**. Upregulation of *Klf2* and *Klf4* in *Ccm3* KO mBMEC. Cells were treated as in A, and an RT-qPCR for *Klf2* (left graph) and *Klf4* (right graph) was performed from *Ccm3* WT and KO cells. mRNA levels relative to *Gapdh* mRNA were normalized to those of *Ccm3* WT cells of each preparation. Graph shows the average and SEM of mRNA relative expression and the individual values of 9 independent biological replicates (cell cultures) for *Klf2* and 8 replicates for *Klf4*. Each cell culture was obtained from a pool of 3 to 4 brains. P-Value from two-sample, two-tailed Student’s t test. **D**. Redistribution of VE-cadherin and β-catenin in *Ccm3* KO mBMEC. Cells were treated as in A and an immunofluorescence performed for β-catenin or VE-cadherin, counterstaining with DAPI. Left panel: representative photographs of the immunofluorescence with staining for VE-cadherin or β-catenin (green) and DNA (blue). Scale bar is 50 µm. Graphs on right show the ratio of junctional/total β-catenin (upper graph) or VE-cadherin (lower graph) after each treatment, referenced to *Ccm3* WT mBMEC of >50 cells per treatment from 2 independent biological replicates (cell cultures). Each cell culture was obtained from a pool of 3 to 4 brains. P-values are from ANOVA analysis with a Tukey’s multiple comparison test.


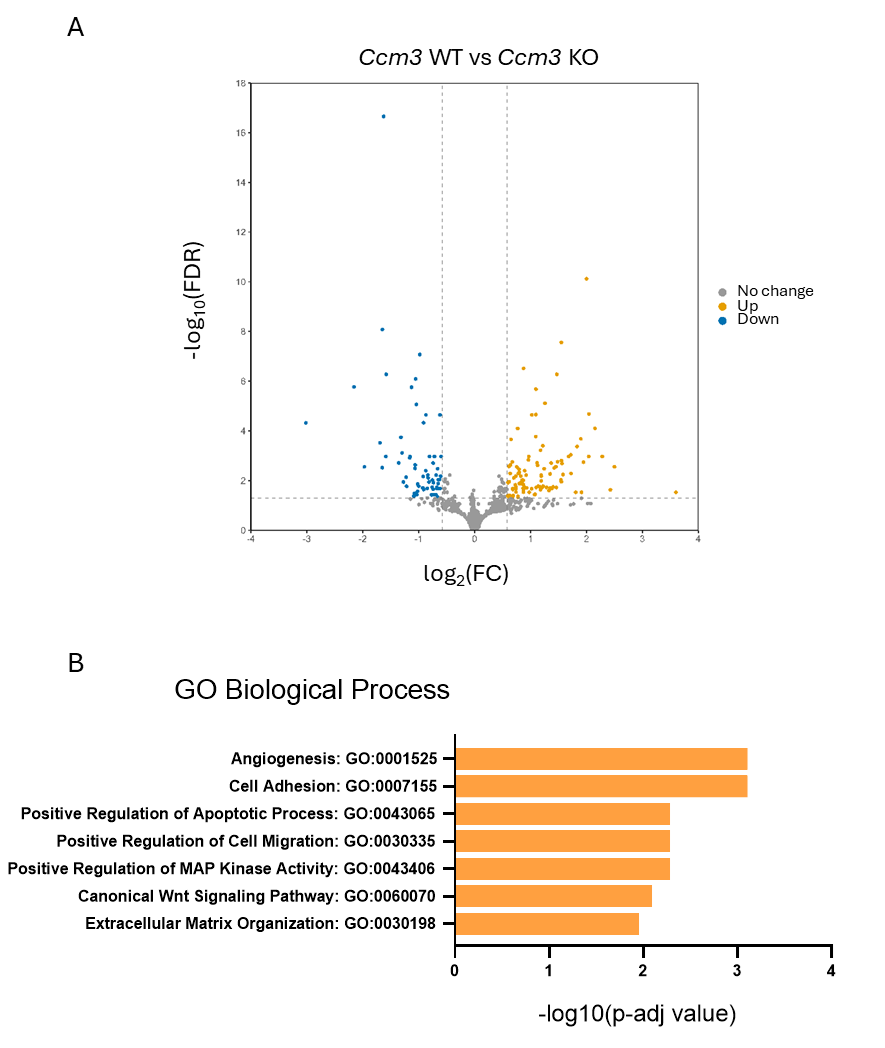


**Figure S2**: **A.** Volcano plot displaying the DEGs between *Ccm3* WT and *Ccm3* KO samples. Genes significantly upregulated or downregulated in KO compared to WT (KO/WT), with FDR < 0.05 and fold change (FC) > 1.5, are shown in yellow (Up) and blue (Down), respectively. Genes not meeting these thresholds are depicted in grey (No change). **B.** Gene ontology analysis of biological process of Ccm3 WT vs KO differentially expressed genes.


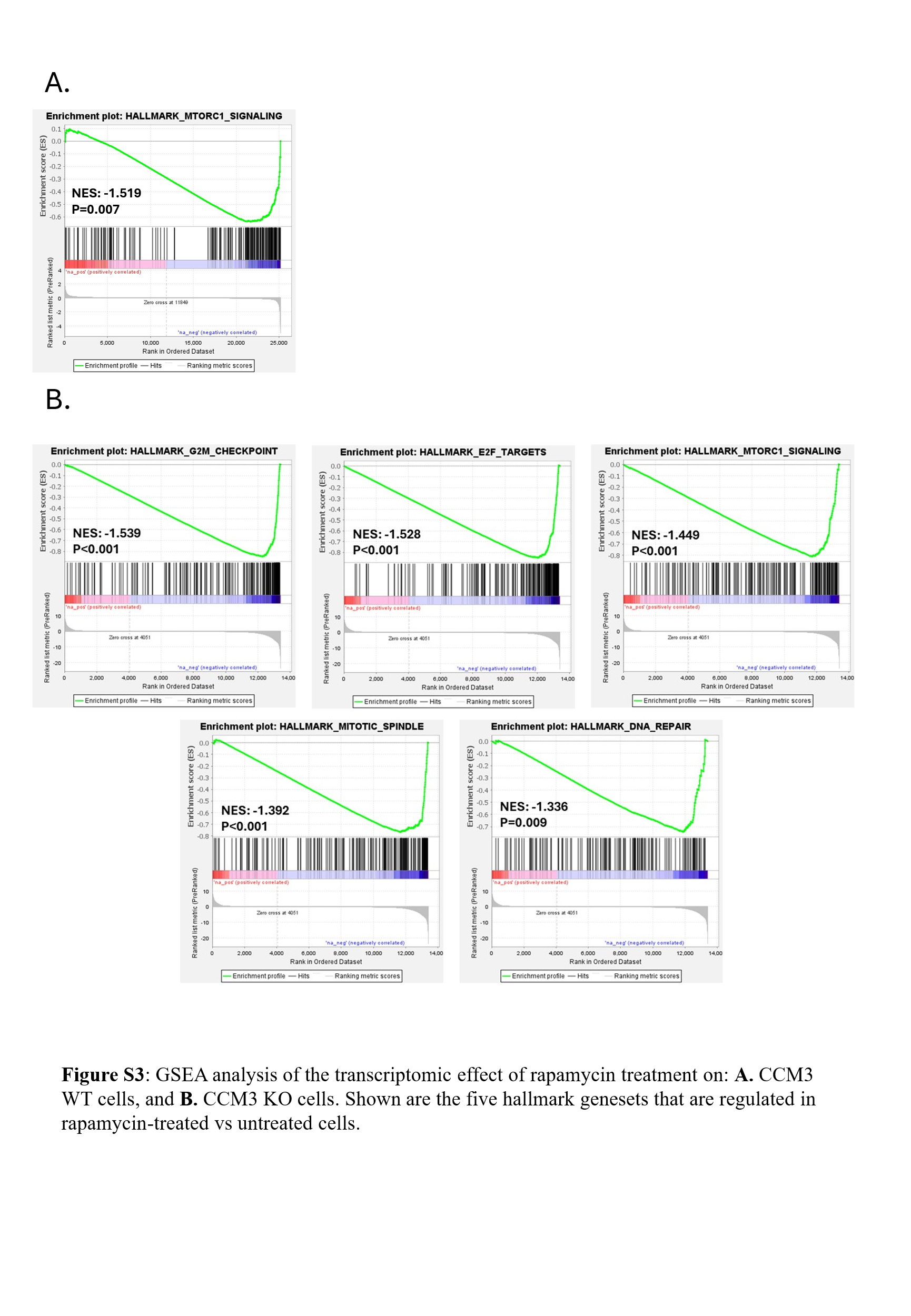


**Figure S3**: GSEA analysis of the transcriptomic effect of rapamycin treatment on: **A.** *Ccm3* WT cells, and **B.** *Ccm3* KO cells. Shown are the hallmark gene sets that are enriched in rapamycin-treated vs untreated cells *Ccm3* KO mBMEC at a nominal p<0.01.


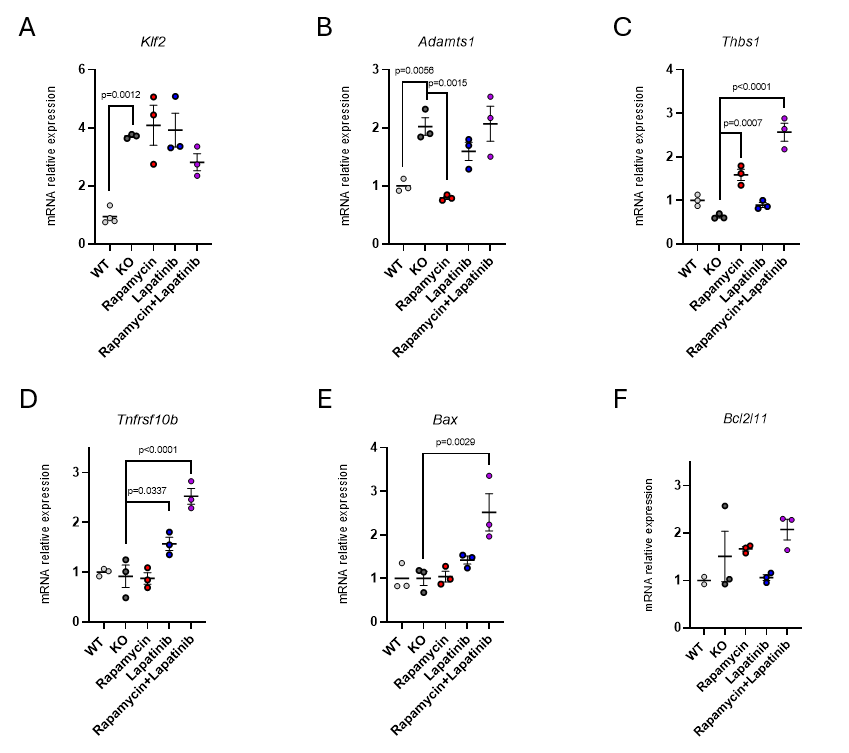


**Figure S4.** Lapatinib induces proapoptotic genes without affecting *Klf2/4*-dependent expression. WT or KO mBMEC were either left untreated or treated with 100 nM rapamycin, 10 μM lapatinib or a combination of both for 24 h. RNA was extracted and RT-qPCR was performed using GAPDH as a reference gene. Each graph shows the average and SEM of mRNA relative expression and the individual values of biological replicates (cell cultures), referenced to the levels of untreated WT mBMEC of the same cell culture. Each cell culture was obtained from a pool of 3 to 4 brains. P-values are from two-way ANOVA analysis with a Tukey’s multiple comparison test. **A.** Expression of *Klf2*. **B.** Expression of *Adamts1*. **C.** Expression of *Thbs1*. **D.** Expression of *Tnfrsf10b.* **E.** Expression of *Bax*. **F.** Expression of *Bcl2l11*.

**Table S1**: Primary antibodies used for immunofluorescence or western blots

| **Antigen** | **Supplier** | **Catalog number** | **Technique** |
| --- | --- | --- | --- |
| VE-cadherin | Abcam | ab205336 | Immunofluorescence |
| b-catenin | BD Biosciences | 610153 | Immunofluorescence |
| GM130 | cell signaling technology | 70767 | Immunofluorescence |
| pS473-AKT | cell signaling technology | 9271 | Western blot |
| AKT | cell signaling technology | 2920 | Western blot |
| CCM3 | Proteintech | 66440 | Western blot |
| eNOS | BD Biosciences | 610297 | Western blot |
| pS1177-eNOS | BD Biosciences | 612393 | Western blot |
| GAPDH | Santa Cruz Biotechnology | sc-47724 | Western blot |
| KLF2 | Proteintech | 23384-1 | Western blot |
| pS371-S6K1 | cell signaling technology | 9208T | Western blot |
| S6K1 | cell signaling technology | 9202S | Western blot |
| Vinculin | Merck Life sciences | V4505 | Western blot |

Table S2: Oligonucleotides used for qPCR

| **Gene** | **Species** | **Forward/reverse** | **Sequence** |
| --- | --- | --- | --- |
| *Gapdh* | Mouse | forward | AGGTCGGTGTGAACGGATTTG |
| *Gapdh* | Mouse | reverse | GGGGTCGTTGATGGCAACA |
| *β-Actin* | Mouse | forward | GTGACGTTGACATCCGTAAAGA |
| *β-Actin* | Mouse | reverse | GCCGGACTCATCGTACTCC |
| *Klf4* | Mouse | forward | GTGCCCCGACTAACCGTTG |
| *Klf4* | Mouse | reverse | GTCGTTGAACTCCTCGGTCT |
| *Klf2* | Mouse | forward | CTCAGCGAGCCTATCTTGCC |
| *Klf2* | Mouse | reverse | CACGTTGTTTAGGTCCTCATCC |
| *Nos3* | Mouse | forward | TGTGACCCTCACCGCTACAA |
| *Nos3* | Mouse | reverse | GCACAATCCAGGCCCAATC |
| *Ccm3* | Mouse | forward | TCACCGAGTCCCTCCTTCG |
| *Ccm3* | Mouse | reverse | GCCCGTGCCTTTTCATTTAGG |
| *Thbs1* | Mouse | forward | CCTGCCAGGGAAGCAACAA |
| *Thbs1* | Mouse | reverse | ACAGTCTATGTAGAGTTGAGCCC |
| *Adamts1* | Mouse | forward | AAGGAAGAAGCGATTTGTGTCC |
| *Adamts1* | Mouse | reverse | CCACCGAGAACAGGGTTAGA |
| *Dll4* | Mouse | forward | TTCCAGGCAACCTTCTCCGA |
| *Dll4* | Mouse | reverse | ACTGCCGCTATTCTTGTCCC |
| *Dll1* | Mouse | forward | GCAGGACCTTCTTTCGCGTAT |
| *Dll1* | Mouse | reverse | AAGGGGAATCGGATGGGGTT |
| *Bcl2l11* | Mouse | forward | CCCGGAGATACGGATTGCAC |
| *Bcl2l11* | Mouse | reverse | GCCTCGCGGTAATCATTTGC |
| *Bax* | Mouse | forward | AGACAGGGGCCTTTTTGCTAC |
| *Bax* | Mouse | reverse | AATTCGCCGGAGACACTCG |
| *Tnfrsf10b* | Mouse | forward | CGGGCAGATCACTACACCC |
| *Tnfrsf10b* | Mouse | reverse | TGTTACTGGAACAAAGACAGCC |

Table S3: *Ccm3* KO vs *Ccm3* WT differentially expressed genes. Effect of propranolol (KOP) or Rapamycin (KOR) treatment in *Ccm3* KO cells

| **GeneSymbol** | **baseMean** | **log2FCKOvsWT** | **pvalue** | **FDR** | **log2KOPvsKO** | **log2KORvsKO** |
| --- | --- | --- | --- | --- | --- | --- |
| *Ltb* | 62.726 | -3.016 | 0.000 | 0.000 | -0.558 | 3.037 |
| *Kirrel3* | 170.813 | -2.156 | 0.000 | 0.000 | -1.396 | 1.756 |
| *Prdm8* | 26.881 | -1.971 | 0.000 | 0.003 | -0.356 | -0.498 |
| *Abca9* | 113.814 | -1.691 | 0.000 | 0.000 | -1.354 | -0.284 |
| *Ptx3* | 421.276 | -1.650 | 0.000 | 0.003 | -0.618 | 0.855 |
| *Six1* | 268.166 | -1.648 | 0.000 | 0.000 | -0.129 | 0.333 |
| *Id3* | 1595.730 | -1.626 | 0.000 | 0.000 | -0.014 | 3.354 |
| *Slco1a5* | 304.361 | -1.588 | 0.000 | 0.001 | 0.148 | 2.163 |
| *Glipr2* | 854.087 | -1.582 | 0.000 | 0.000 | 0.531 | -0.804 |
| *Bmp2* | 3187.518 | -1.356 | 0.000 | 0.002 | 1.390 | -3.049 |
| *Adora2a* | 303.368 | -1.317 | 0.000 | 0.000 | -0.793 | -2.976 |
| *Edn1* | 7918.723 | -1.296 | 0.000 | 0.001 | -0.121 | 2.091 |
| *Bdnf* | 147.357 | -1.272 | 0.000 | 0.011 | 0.167 | 1.903 |
| *Cfh* | 118.005 | -1.223 | 0.000 | 0.007 | -0.698 | 0.513 |
| *Egln3* | 1474.220 | -1.217 | 0.000 | 0.017 | -1.414 | -0.049 |
| *Ankrd1* | 2001.968 | -1.161 | 0.000 | 0.001 | 1.225 | 1.567 |
| *Pfkfb3* | 1929.920 | -1.154 | 0.000 | 0.001 | -0.669 | 0.701 |
| *Lamb1* | 7399.706 | -1.129 | 0.000 | 0.000 | -0.066 | 0.217 |
| *Cdkl1* | 182.071 | -1.089 | 0.001 | 0.042 | -1.373 | -1.384 |
| *Gm17501* | 145.655 | -1.079 | 0.000 | 0.033 | 1.571 | 2.061 |
| *Pdcd10* | 514.510 | -1.072 | 0.001 | 0.039 | 0.510 | -0.275 |
| *Gna14* | 172.007 | -1.064 | 0.000 | 0.003 | -0.469 | -0.639 |
| *Cpm* | 151.257 | -1.062 | 0.000 | 0.002 | -0.698 | 0.136 |
| *Pcdh7* | 809.810 | -1.056 | 0.000 | 0.000 | -0.411 | 0.941 |
| *Ilvbl* | 1355.747 | -1.040 | 0.000 | 0.000 | -0.096 | -0.211 |
| *Col27a1* | 124.740 | -1.031 | 0.000 | 0.037 | -0.649 | 0.272 |
| *Prnd* | 1517.553 | -1.022 | 0.000 | 0.027 | -0.899 | 0.742 |
| *Pim3* | 4036.250 | -1.019 | 0.000 | 0.014 | -0.457 | 1.411 |
| *Mmp25* | 1414.248 | -0.998 | 0.000 | 0.017 | -0.915 | -0.325 |
| *2610008E11Rik* | 2021.129 | -0.980 | 0.000 | 0.000 | -0.777 | 0.581 |
| *Mafb* | 281.945 | -0.926 | 0.000 | 0.020 | -0.884 | 0.970 |
| *Zic3* | 1168.382 | -0.918 | 0.000 | 0.007 | -0.406 | 1.669 |
| *Ccn1* | 17206.153 | -0.912 | 0.000 | 0.000 | 0.393 | 1.672 |
| *Adm* | 12819.649 | -0.910 | 0.000 | 0.023 | -0.219 | 0.384 |
| *Dpysl3* | 6298.625 | -0.874 | 0.000 | 0.004 | 0.119 | -0.395 |
| *Thbs1* | 60206.476 | -0.871 | 0.000 | 0.000 | -0.124 | 1.296 |
| *Dapk2* | 1670.080 | -0.845 | 0.000 | 0.021 | -0.523 | -2.155 |
| *Vgf* | 376.075 | -0.838 | 0.000 | 0.006 | 0.767 | 0.524 |
| *Nox4* | 9553.510 | -0.831 | 0.000 | 0.008 | -0.295 | -0.393 |
| *Ppp1r3b* | 243.953 | -0.815 | 0.000 | 0.011 | 0.007 | 0.380 |
| *Fblim1* | 1546.851 | -0.806 | 0.000 | 0.001 | -0.765 | 0.891 |
| *Cdh2* | 1034.499 | -0.770 | 0.000 | 0.038 | -0.495 | 0.024 |
| *4930555A03Rik* | 111.828 | -0.760 | 0.000 | 0.018 | -0.408 | 0.148 |
| *Ccn2* | 32489.278 | -0.755 | 0.000 | 0.010 | 0.596 | 1.820 |
| *Usp53* | 581.139 | -0.742 | 0.000 | 0.022 | -0.213 | 0.502 |
| *Sgk1* | 2823.181 | -0.742 | 0.000 | 0.002 | -0.120 | 1.353 |
| *Ppp1r37* | 7620.383 | -0.738 | 0.000 | 0.037 | -0.219 | 0.093 |
| *Tnc* | 9913.686 | -0.736 | 0.000 | 0.012 | 0.086 | -0.110 |
| *Kctd11* | 1011.621 | -0.719 | 0.000 | 0.001 | -0.043 | -0.405 |
| *Fzd1* | 792.231 | -0.694 | 0.000 | 0.037 | -0.494 | 0.066 |
| *Prrg3* | 509.990 | -0.687 | 0.000 | 0.006 | -0.353 | -0.335 |
| *Lratd2* | 199.339 | -0.677 | 0.000 | 0.021 | 0.177 | -0.173 |
| *Tspan6* | 4564.825 | -0.676 | 0.000 | 0.019 | -0.418 | 0.303 |
| *Colec12* | 1846.702 | -0.671 | 0.000 | 0.021 | -0.901 | -0.428 |
| *Dach1* | 765.308 | -0.661 | 0.000 | 0.003 | -0.117 | -0.540 |
| *Wnt9a* | 1206.375 | -0.661 | 0.001 | 0.046 | -0.386 | -0.788 |
| *Unc5b* | 3060.077 | -0.659 | 0.000 | 0.021 | -0.906 | -0.215 |
| *Sp5* | 1165.472 | -0.650 | 0.000 | 0.013 | 0.275 | 1.878 |
| *Arhgef37* | 278.231 | -0.647 | 0.000 | 0.009 | -0.951 | 0.344 |
| *Col15a1* | 1332.740 | -0.626 | 0.000 | 0.009 | -0.808 | 1.108 |
| *Csrp2* | 2230.716 | -0.620 | 0.000 | 0.000 | -0.583 | 1.557 |
| *Pawr* | 430.348 | -0.616 | 0.000 | 0.021 | 0.015 | 1.585 |
| *Frzb* | 4416.046 | -0.608 | 0.000 | 0.007 | -0.689 | 0.936 |
| *Ctps* | 5456.071 | -0.599 | 0.000 | 0.001 | -0.417 | 1.125 |
| *Fam83g* | 186.777 | 0.594 | 0.001 | 0.040 | 0.489 | -0.294 |
| *Lgals3* | 1121.020 | 0.597 | 0.001 | 0.043 | 1.588 | -0.812 |
| *Ncoa3* | 1399.829 | 0.600 | 0.000 | 0.012 | 0.001 | -0.289 |
| *Slc9a3r2* | 18386.962 | 0.600 | 0.000 | 0.011 | -0.367 | -0.269 |
| *Tpcn1* | 3077.855 | 0.617 | 0.000 | 0.003 | -0.387 | -0.468 |
| *Ephb4* | 3510.055 | 0.619 | 0.000 | 0.011 | -0.551 | -0.221 |
| *Hsph1* | 4516.268 | 0.626 | 0.001 | 0.040 | -0.284 | -0.364 |
| *Gbp9* | 841.018 | 0.636 | 0.000 | 0.005 | -1.251 | -1.241 |
| *Slc30a1* | 1844.929 | 0.643 | 0.000 | 0.002 | 0.386 | -0.447 |
| *Atp8a1* | 886.045 | 0.652 | 0.000 | 0.000 | -0.082 | -0.150 |
| *Sec14l1* | 8976.631 | 0.675 | 0.000 | 0.002 | 0.156 | -0.173 |
| *Baiap2l1* | 265.811 | 0.682 | 0.001 | 0.045 | 0.664 | -0.124 |
| *C2cd2l* | 1933.500 | 0.684 | 0.000 | 0.021 | -0.140 | -0.615 |
| *Cbfa2t3* | 1280.891 | 0.685 | 0.000 | 0.023 | -0.539 | -0.396 |
| *Nt5dc2* | 400.140 | 0.690 | 0.001 | 0.040 | -0.289 | -1.345 |
| *Itprip* | 785.427 | 0.693 | 0.000 | 0.021 | 1.136 | -0.190 |
| *Kcnq1* | 1296.399 | 0.701 | 0.000 | 0.008 | 0.551 | -0.906 |
| *Ms4a6d* | 508.791 | 0.729 | 0.000 | 0.015 | -0.158 | -0.591 |
| *Sema3g* | 1472.637 | 0.739 | 0.000 | 0.017 | -0.733 | -0.441 |
| *Lama5* | 6526.196 | 0.740 | 0.000 | 0.007 | -0.453 | 0.183 |
| *Plpp3* | 1372.492 | 0.740 | 0.000 | 0.029 | -0.492 | -0.692 |
| *Zbtb46* | 499.783 | 0.741 | 0.000 | 0.021 | 0.031 | -0.064 |
| *Palmd* | 1555.168 | 0.756 | 0.000 | 0.003 | 0.672 | 0.523 |
| *Pcdh12* | 1064.533 | 0.770 | 0.000 | 0.000 | -1.597 | -2.847 |
| *Abi3* | 490.846 | 0.774 | 0.001 | 0.043 | -0.485 | -0.322 |
| *Rps6kl1* | 414.087 | 0.775 | 0.001 | 0.047 | -0.106 | -0.745 |
| *Mylip* | 371.901 | 0.783 | 0.000 | 0.005 | -0.218 | 0.023 |
| *Prodh* | 373.409 | 0.794 | 0.000 | 0.004 | -0.283 | 0.082 |
| *Gsdmd* | 901.737 | 0.797 | 0.000 | 0.013 | -0.211 | -0.204 |
| *Arhgef15* | 2354.556 | 0.801 | 0.000 | 0.005 | -0.283 | -0.372 |
| *Zfp697* | 1023.276 | 0.828 | 0.000 | 0.007 | 0.745 | -0.175 |
| *Tsc22d3* | 684.771 | 0.834 | 0.000 | 0.013 | 0.721 | -0.654 |
| *Dhcr24* | 1106.425 | 0.843 | 0.000 | 0.011 | 0.873 | -0.278 |
| *Rtl8c* | 357.129 | 0.849 | 0.000 | 0.010 | -0.280 | -0.386 |
| *Jag2* | 3543.888 | 0.855 | 0.000 | 0.009 | -0.044 | -0.782 |
| *Ccm2l* | 2256.064 | 0.868 | 0.000 | 0.020 | -0.676 | -0.352 |
| *H2-T23* | 1374.304 | 0.870 | 0.000 | 0.010 | -0.302 | -0.632 |
| *Apold1* | 8841.068 | 0.871 | 0.000 | 0.014 | 1.005 | -0.773 |
| *Rtl8b* | 1883.415 | 0.872 | 0.000 | 0.023 | 0.004 | -0.608 |
| *Adamts1* | 3885.692 | 0.874 | 0.000 | 0.029 | 0.627 | -1.814 |
| *Slfn5* | 6589.197 | 0.877 | 0.000 | 0.000 | -0.303 | -0.033 |
| *Dok4* | 1071.393 | 0.885 | 0.000 | 0.017 | -0.630 | -0.905 |
| *Als2cl* | 1580.216 | 0.894 | 0.000 | 0.004 | -0.505 | 0.041 |
| *Efemp2* | 549.492 | 0.920 | 0.000 | 0.006 | -0.450 | -0.086 |
| *Tmc8* | 156.790 | 0.929 | 0.000 | 0.020 | 0.001 | -0.939 |
| *Pik3r6* | 419.433 | 0.962 | 0.000 | 0.001 | -0.723 | -0.802 |
| *Synm* | 1197.688 | 0.970 | 0.000 | 0.001 | 0.117 | 0.387 |
| *Hspa1b* | 202.184 | 0.976 | 0.000 | 0.030 | 1.514 | -1.208 |
| *Gatm* | 189.326 | 1.020 | 0.000 | 0.021 | -0.102 | -1.373 |
| *Aatk* | 491.670 | 1.023 | 0.000 | 0.000 | -0.613 | -0.355 |
| *Gm41442* | 1639.899 | 1.075 | 0.000 | 0.036 | -0.108 | -0.473 |
| *Alas1* | 9658.050 | 1.084 | 0.000 | 0.035 | -0.544 | 0.873 |
| *Scd2* | 6279.980 | 1.094 | 0.000 | 0.000 | 1.257 | -1.119 |
| *Mmp15* | 568.496 | 1.096 | 0.000 | 0.000 | -0.977 | -2.019 |
| *Sh3tc2* | 730.908 | 1.098 | 0.000 | 0.000 | -0.479 | -1.389 |
| *Podxl* | 28731.251 | 1.102 | 0.000 | 0.019 | 0.163 | -0.376 |
| *Fam167a* | 880.767 | 1.118 | 0.000 | 0.002 | 0.670 | -1.390 |
| *Fam20a* | 414.381 | 1.126 | 0.000 | 0.002 | 0.097 | -1.107 |
| *Adgre5* | 2838.583 | 1.137 | 0.000 | 0.017 | -0.821 | -0.444 |
| *Magix* | 53.304 | 1.159 | 0.000 | 0.017 | -0.362 | 0.357 |
| *Lsr* | 809.203 | 1.178 | 0.000 | 0.023 | -0.296 | -0.452 |
| *Sema7a* | 3331.687 | 1.180 | 0.000 | 0.001 | -0.409 | -0.642 |
| *Efr3b* | 281.288 | 1.190 | 0.000 | 0.006 | 0.907 | -0.648 |
| *Ramp2* | 10575.180 | 1.193 | 0.000 | 0.010 | -0.486 | -0.843 |
| *Ecm1* | 6559.986 | 1.194 | 0.000 | 0.007 | -0.545 | -0.719 |
| *Rtl8a* | 2573.965 | 1.202 | 0.000 | 0.019 | 0.146 | -0.989 |
| *Ckb* | 3249.226 | 1.221 | 0.000 | 0.000 | -0.502 | -0.999 |
| *Klf4* | 3046.533 | 1.225 | 0.000 | 0.017 | 1.049 | -0.175 |
| *Zswim1* | 1126.193 | 1.244 | 0.000 | 0.003 | 0.065 | 0.110 |
| *Ccn3* | 438.764 | 1.259 | 0.000 | 0.000 | 0.764 | 1.900 |
| *Nkd2* | 282.131 | 1.286 | 0.000 | 0.019 | 0.047 | -0.694 |
| *Afap1l2* | 270.406 | 1.331 | 0.000 | 0.025 | -0.323 | -0.418 |
| *Irx3* | 321.497 | 1.349 | 0.000 | 0.020 | -0.071 | 0.273 |
| *Endou* | 137.096 | 1.350 | 0.000 | 0.005 | 0.247 | -0.450 |
| *Gbp4* | 341.962 | 1.369 | 0.000 | 0.002 | -1.327 | -2.236 |
| *Rd3* | 48.516 | 1.380 | 0.000 | 0.021 | -0.239 | -0.110 |
| *Nr4a1* | 1078.578 | 1.402 | 0.000 | 0.018 | 1.764 | -0.524 |
| *Mcoln2* | 249.784 | 1.426 | 0.000 | 0.003 | 0.858 | -2.115 |
| *Nkd1* | 76.919 | 1.454 | 0.000 | 0.003 | -0.386 | -0.615 |
| *Ptprr* | 388.445 | 1.462 | 0.000 | 0.019 | 1.058 | -0.233 |
| *Jam2* | 260.413 | 1.470 | 0.000 | 0.000 | -0.126 | -0.850 |
| *Ankrd33b* | 536.405 | 1.485 | 0.000 | 0.002 | -0.379 | -0.612 |
| *Syt7* | 72.791 | 1.545 | 0.000 | 0.009 | -0.694 | -0.979 |
| *Kctd12* | 935.486 | 1.551 | 0.000 | 0.000 | -0.410 | -0.664 |
| *Ip6k3* | 143.725 | 1.552 | 0.000 | 0.011 | -0.480 | -1.371 |
| *Pltp* | 31156.884 | 1.552 | 0.000 | 0.002 | -0.278 | 0.272 |
| *Ceacam1* | 999.617 | 1.563 | 0.000 | 0.002 | -0.302 | 0.129 |
| *Dhrs3* | 72.001 | 1.581 | 0.000 | 0.006 | -0.663 | -1.205 |
| *Nqo1* | 1745.515 | 1.681 | 0.000 | 0.001 | 0.256 | -1.136 |
| *Neurl2* | 1448.766 | 1.719 | 0.000 | 0.001 | -0.058 | 0.118 |
| *Nostrin* | 66.065 | 1.724 | 0.000 | 0.005 | 0.017 | -1.137 |
| *Slco2a1* | 86.304 | 1.809 | 0.000 | 0.030 | 0.077 | -1.277 |
| *Anpep* | 548.541 | 1.832 | 0.000 | 0.000 | 0.002 | -1.534 |
| *Apod* | 14346.608 | 1.900 | 0.000 | 0.000 | -0.127 | -1.275 |
| *Sox11* | 25.532 | 1.913 | 0.000 | 0.030 | 3.984 | -2.370 |
| *Ednrb* | 243.824 | 1.947 | 0.000 | 0.002 | 1.131 | -2.453 |
| *Pcp4l1* | 294.594 | 2.002 | 0.000 | 0.000 | -0.033 | -0.400 |
| *Rtp3* | 99.712 | 2.042 | 0.000 | 0.001 | -0.616 | -1.550 |
| *Clic5* | 876.559 | 2.045 | 0.000 | 0.000 | 0.502 | -0.635 |
| *Xdh* | 171.846 | 2.152 | 0.000 | 0.000 | -0.445 | 0.345 |
| *Car5a* | 32.536 | 2.283 | 0.000 | 0.001 | 1.680 | -0.538 |
| *Wnt9b* | 26.699 | 2.429 | 0.000 | 0.023 | 0.166 | -1.868 |
| *Spata25* | 137.464 | 2.502 | 0.000 | 0.003 | -0.212 | 0.297 |
| *Srarp* | 11.079 | 3.600 | 0.000 | 0.030 | -0.968 | -1.902 |
